# Potential Causal Association between Depression and Oral Diseases: A Mendelian Randomization Study

**DOI:** 10.3390/genes14122191

**Published:** 2023-12-08

**Authors:** Xiaobin Zhang, Hehe Jiang, Linlin Zhang, Chunjing Li, Chen Chen, Mengzhen Xing, Yuning Ma, Yuxia Ma

**Affiliations:** 1 College of Acupuncture, Moxibustion and Tuina, Shandong University of Traditional Chinese Medicine, Jinan 250355, China; 2021100056@sdutcm.edu.cn (X.Z.); 60050067@sdutcm.edu.cn (C.L.); phdmayuxia@126.com (Y.M.); 2 Institute of Pharmacy, Shandong University of Traditional Chinese Medicine, Jinan 250355, China; 18396852678@163.com (H.J.); linlin66210@outlook.com (L.Z.); 21129008@zju.edu.cn (C.C.); mengzhen@mail.ipc.ac.cn (M.X.)

**Keywords:** causal relationship, depression, oral diseases, Mendelian randomization analysis, genome-wide association studies

## Abstract

Background: Globally, oral diseases are common, pose an economic burden, and significantly decline the quality of life of affected individuals. Recently, researchers have substantially highlighted the effect of depression on oral disease incidence and development. In this study, we elucidated the correlation between depression and oral diseases. Methods: Using two-sample Mendelian randomization (MR), the association between depression and the risk of 17 oral diseases was evaluated. Three methods were used to perform MR analysis: the inverse variance-weighted, weighted median, and MR-Egger methods. Furthermore, Cochran’s Q test, MR-Egger intercept test, MR Pleiotropy RESidual Sum and Outlier test, and leave-one-out analysis were performed to analyze sensitivity. Results: After implementing multiple test corrections, we observed that genetic susceptibility to depression was associated with an increased risk of mouth ulcers, toothache, loose teeth, bleeding gums, painful gums, chronic periodontitis, chronic tonsil and adenoid diseases, peritonsillar abscess, and excessive tooth attrition. However, a causal relationship between depression and other oral diseases was not observed. Sensitivity analysis confirmed the robustness of the results. Conclusions: We confirmed the causal relationship between depression and several oral diseases, thereby providing a novel viewpoint on the prevention and treatment of oral diseases. Our findings suggest the integration of depression control into routine clinical care to enhance the effectiveness of oral disease treatment.

## 1. Introduction

Depression, a common mood disorder, is characterized by persistent melancholy, debilitatingly low mood, cognitive impairment, and loss of interest [1]. In serious situations, depression is associated with an increased risk of suicidal thoughts, suicide attempts, and even death [2]. The morbidity, recurrence, and disability rates of depression are high [3]. As per the World Health Organization (WHO) estimates, 5% of the adult population worldwide suffers from depression [4]. By 2030, depression will become the leading reason for disease burden globally [5]. Because depression negatively affects both mental and physical health, it has emerged as a significant public health issue.

Various variables, including local conditions, systemic health, behavioral patterns, and psychological factors, may contribute to disease development [6]. Depression may increase the risk of susceptibility to various medical conditions in the future. With an increase in depression prevalence, its effect on oral health is garnering attention. Alcohol consumption and smoking are both considered risk factors for developing depression and oral diseases [7]. Nevertheless, the effect of depression on diseases may be partially alleviated by these and other behaviors such as noncompliance with dental treatment and difficulties in attaining dental services and maintaining proper oral hygiene [8]. Sugar consumption is closely associated with oral health issues. Excessive sugar consumption markedly increases the risk of developing oral diseases, including dental caries, particularly among children and adolescents [9,10]. Many studies have revealed that removable prostheses can lead to chronic atrophic candidiasis, periodontitis, and stomatitis because of the presence of many interfaces at which microbes can adhere and form plaque [11,12]. Furthermore, vitamin A, B1, B2, C, D, and E levels are strongly correlated with a significant risk of developing oral diseases, including periodontitis and tooth loss. However, oral health can be improved by maintaining appropriate vitamin levels [13,14]. Despite notable advances in oral health development in many countries, oral diseases remain a worldwide challenge as per a review issued by the WHO [15]. In addition, the crucial nature of oral health is collectively underscored by the detrimental effects of oral diseases on speech, appearance, biting, and financial strain [16]. Therefore, comprehensively understanding the approaches by which better results can be achieved in treating oral diseases is vital. A collection of observational studies have revealed that people with depression are more likely to develop mouth ulcers [17] and that a genetic correlation exists between depression and mouth ulcers [18]. Furthermore, studies suggest that chronic stress is significantly associated with plaque and that long-term exposure to chronic strain, which results in changes in salivary flow rate, pondus hydrogenii (pH), and the levels of components such as salivary cortisol, can directly or indirectly promote plaque accumulation; this leads to poor oral hygiene and the increased incidence of caries, periodontal disease, and plaque-related diseases [19,20,21,22]. Therefore, the abovementioned observations support the hypothesis that depression can lead to oral diseases. However, observational studies are prone to biases, including the presence of unmeasured confounding variables. For example, ketamine, a commonly prescribed medication for depression, markedly increases cortisol production, thereby interfering with oral status assessment [23,24]. Hence, the relationship between depression and oral diseases remains uninvestigated systematically; furthermore, whether depression casually contributes to oral disease onset remains unclear owing to potential biases in previous observational studies.

Mendelian randomization (MR) is a statistical method to determine the causal relationship between exposures and outcomes by using genetic variants as instrumental variables (IVs) [25]. Because the genotypes are randomly assigned from parents to progeny, the association between genetic variants and outcomes is not affected by conventional confounding variables and reverse causation; therefore, a causal relationship may be obtained [26]. MR is widely used to investigate disease pathogenesis [27]. Many studies that have utilized MR methods have revealed that depression is significantly correlated with many diseases, including prostate cancer, inflammatory bowel disease, osteoporosis, and gastrointestinal diseases [28,29,30,31]. Therefore, based on the remarkable dependability of previous MR investigations in establishing causation, in this study, we elucidated the correlation between depression and oral diseases using different MR methods. Through this investigation, we hope to provide fresh empirical support for research advances in this domain.

## 2. Methods

### 2.1. Study Design

**Figure 1:**
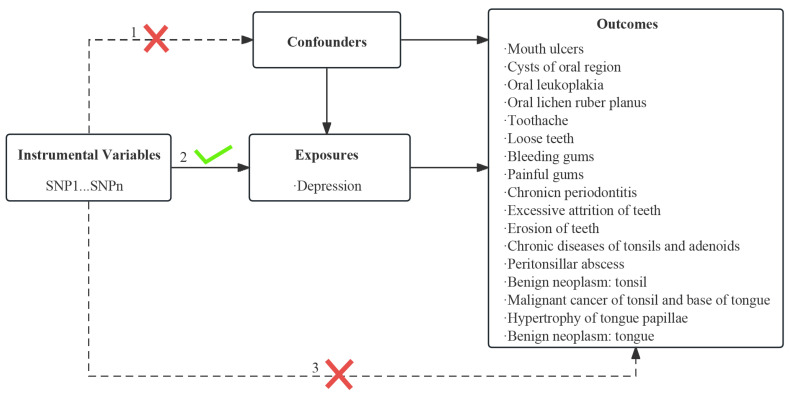
Three hypotheses underpin the Mendelian randomization study: 1. the independence of instrumental variables from confounding factors; 2. the instrumental variables has a close relationship with exposure; 3. instrumental variables exclusively influence outcomes through exposure, other than through any other way.

Using the summary statistics of genome-wide association studies (GWASs), a two-sample MR analysis was performed to elucidate the causal association between depression and oral diseases. To obtain unbiased causal effects, the MR analysis should adhere to the following three presumptions: (1) genetic variants are strongly associated with the exposure of interest; (2) genetic variants are not correlated with potential confounders; and (3) genetic variants affect outcomes only via the exposure of interest. Additional ethical approval was not needed to reanalyze the previously collected and published data. This MR study aimed to fulfill the three primary assumptions explained in Figure 1.

### 2.2. Deriving Genetic Instruments for Depression

The most recent GWASs were used to extract genetic IVs for depression. These GWASs had meta-analyzed the data of 807,553 individuals (246,363 cases and 561,190 controls, all of European ancestry) from the three largest existing genetic studies on depression (the United Kingdom Biobank (UK Biobank) study, 23andMe, and the Psychiatric Genomics Consortium) [32].

### 2.3. Data Source for Oral Diseases

The summary statistics for bleeding gums, loose teeth, toothache, painful gums, and mouth ulcers were obtained from the UK Biobank (http://www.nealelab.is/uk-biobank/, accessed on 21 September 2023), which included datasets for bleeding gums (GWAS ID “ukb-b-7872”), loose teeth (GWAS ID “ukb-b-12849”), toothache (GWAS ID “ukb-b-19191”), painful gums (GWAS ID “ukb-b-11161”), and mouth ulcers (GWAS ID “ukb-b-6458”). The data related to cysts of the oral region (1223 cases and 259,234 controls, all of the European ancestry), oral leukoplakia (474 cases and 376,803 controls, all of European ancestry), oral lichen ruber planus (510 cases and 376,767 controls, all of European ancestry), periodontitis (4434 cases and 259,234 controls, all of European ancestry), excessive tooth attrition (840 cases and 259,234 controls, all of European ancestry), dental erosion (425 cases and 259,234 controls, all of European ancestry), chronic tonsil and adenoid diseases (43,325 cases and 283,342 controls, all of European ancestry), peritonsillar abscess (7510 cases and 283,342 controls, all of European ancestry), benign neoplasm of the tonsil (281 cases and 376,996 controls, all of European ancestry), malignant cancer of the tonsil and base of the tongue (443 cases and 287,137 controls, all of European ancestry), hypertrophy of the tongue papillae (268 cases and 377,009 controls, all of European ancestry), and benign neoplasm of the tongue (720 cases and 376,557 controls, all of European ancestry) were utilized, which were obtained from the European samples of the FinnGen project (https://www.finngen.fi/en, accessed on 21 September 2023) [33]. Table S1 presents detailed information on the oral diseases. The R9 release of the data for the FinnGen study was used; this eliminates individuals with non-Finnish ancestry, ambiguous sex, high genotype missingness (>5%), and high heterozygosity (±4 standard deviation (SD)).

### 2.4. Selection of Genetic Instruments

**Figure 2:**
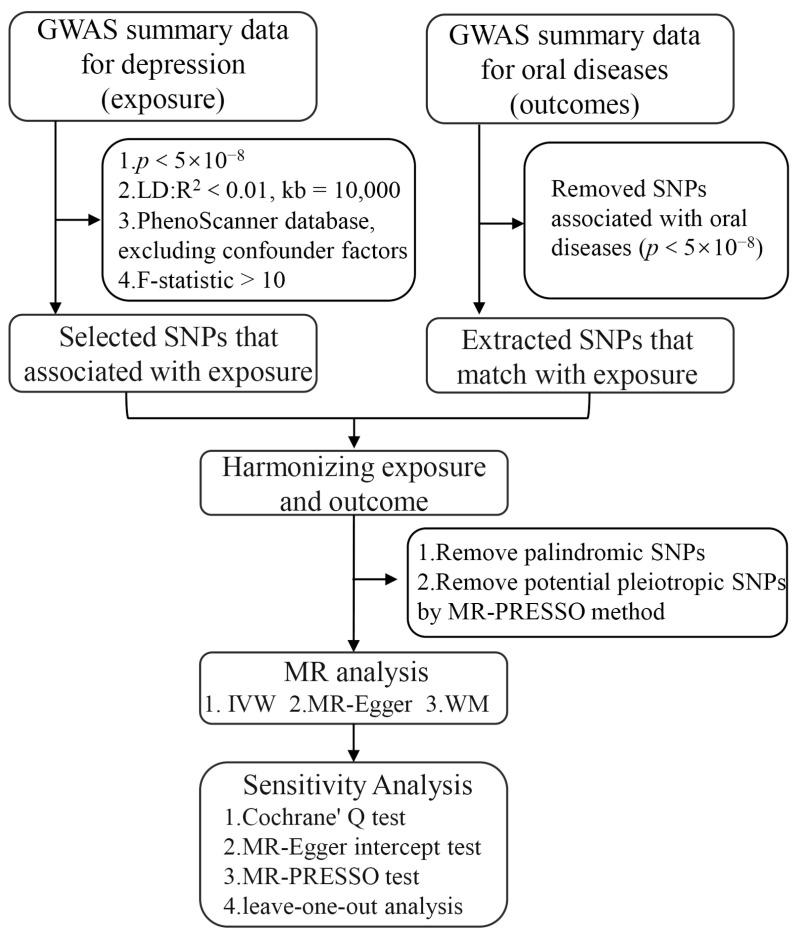
The schematic flow diagram of this study.

To ensure the robustness and dependability of the MR analysis, the IVs that satisfied the three MR analysis assumptions were subjected to various stringent quality controls. First, the single-nucleotide polymorphisms (SNPs) strongly associated with depression (*p* < 5 × 10^−8^) were obtained. In total, 102 distinct independent variants were identified [34,35]. Second, SNPs with a strong linkage disequilibrium (LD) were removed because they could produce biased results (r^2^ < 0.01 and clumping distance = 10,000 kb) [31]. As a result, 98 SNPs were identified. Third, the 98 SNPs associated with depression, which were obtained using the PhenoScanner database (http://www.phenoscanner.medschl.cam.ac.uk/, accessed on 28 September 2023), were searched on a case-by-case basis. The SNPs associated with potential confounders such as alcohol consumption, smoking, sugar consumption, denture wearing, and vitamin A, B1, B2, C, D, and E deficiencies were excluded [13,14,36,37,38,39]. As a result, 96 SNPs were identified. Fourth, SNPs with an F-statistic value > 10 were selected because they are often considered highly likely to be associated with depression [40]. Fifth, the SNPs associated with oral diseases (*p* < 5 × 10^−8^) were excluded [41]. Sixth, palindromic SNPs were removed from the harmonization of GWASs for depression and oral diseases [42]. Next, to overcome potential horizontal pleiotropy, potential outlier SNPs were identified using the MR Pleiotropy RESidual Sum and Outlier (MR-PRESSO) test. Finally, the remaining SNPs were used in the MR analysis. Figure 2 illustrates the flowchart of the study.

## 3. MR Analysis

### Statistical Analysis

The inverse variance-weighted (IVW), weighted median (WM), and MR-Egger methods were used to determine the MR estimates of depression for oral diseases. The primary analysis employed the IVW method with a random effects model, which assumes that IVs may only affect the outcome via exposure [43,44]. When more than 50% of the information is derived from valid IVs, the WM method provides consistent estimates [45]. The hypothesis of the MR-Egger method is that variant–exposure associations are not associated with the pleiotropic effects of the genetic variants [45]. The odds ratio (OR) is the effect magnitude that establishes the causal relationship. An OR value of <1 indicates that the exposure variable functions as a protective factor against the outcome. If the estimations derived from the methods used in this study were inconsistent, a stricter instrument *p*-value criterion was established [46].

In MR studies, sensitivity analysis plays a vital role in detecting underlying pleiotropy. Furthermore, for MR estimates, heterogeneity can be severely violated.

Cochran’s Q statistic (MR-IVW) and Rucker’s Q statistic (MR-Egger) were calculated to determine the heterogeneity of our MR analysis. A *p*-value of <0.05 indicated heterogeneity [47]. However, the presence of heterogeneity does not inherently render the IVW model unreliable [41]. To analyze the robustness of our findings and identify potential horizontal pleiotropy, sensitivity analyses were performed using the MR-Egger intercept test, MR-PRESSO test, and leave-one-out method. The MR-Egger regression intercept suggested the presence of directional pleiotropy (*p* < 0.05 indicated directional pleiotropy) [41]. Furthermore, the MR-PRESSO test was used to detect the outliers associated with horizontal pleiotropy and correct any outlier-induced distortion (NbDistribution = 5000) [48]. In addition, the leave-one-out method was used to determine whether the causal association was driven by a single SNP. In this method, each exposure-related SNP was removed in turn and the IVW analysis was repeated [42].

Statistical analyses were performed using TwoSampleMR (version 0.5.7) and MR-PRESSO (version 1.0) in R (version 4.3.0).

## 4. Results

Information on the research and samples that were used in this study is comprehensively summarized in Table S1. All the included individuals were of European ancestry, and both men and women participated in the research. According to the initially devised screening protocol, 102 independent SNPs from the GWASs on depression were identified as significant (*p* < 5 × 10^−8^) genome-wide [35]. In total, 98 SNPs with an LD of r^2^ > 0.01 and kb = 10,000 were identified [34]. The PhenoScanner database was used to assess whether these SNPs were associated with potential confounders such as alcohol consumption, smoking, sugar consumption, denture wearing, and vitamin A, B1, B2, C, D, and E deficiencies [13,14,36,37,38,39]. When extracting the exposure SNPs from the outcome phenotype of oral diseases, two SNPs, namely, rs17641524 and rs200949, were excluded owing to their strong relationship with oral diseases. The F-statistics for the IVs used for depression were >10, suggesting that weak instrument bias was implausible. Finally, in the follow-up MR analysis, 96 genetic variants that exhibited a significant association with depression were used. Table S2 presents comprehensive details regarding the selected genetic IVs.

When harmonizing depression and 5 of the 17 oral diseases (mouth ulcers, toothache, loose teeth, bleeding gums, and painful gums), 6 palindromic SNPs, namely, rs12052908, rs1933802, rs2029865, rs2247523, rs263645, and rs2876520, were removed because they were absent in the outcome GWASs. When harmonizing depression and 12 of the 17 oral diseases (chronic periodontitis, chronic tonsil and adenoid diseases, peritonsillar abscess, excessive tooth attrition, cysts of the oral region, oral leukoplakia, oral lichen ruber planus, dental erosion, hypertrophy of the tongue papillae, malignant cancer of the tonsil and base of the tongue, benign neoplasm of the tonsil, and benign neoplasm of the tongue), 8 palindromic SNPs, namely, rs10061069, rs12967143, rs1933802, rs2029865, rs2247523, rs263645, rs2876520, and rs7758630, were removed because they were absent in the outcome GWASs.

The MR-PRESSO distortion test identified one outlier (rs10789214) for depression and bleeding gums, and one outlier (rs301799) for depression and chronic tonsil disease in the MR analysis. No outliers were identified for depression and the other oral diseases.

**Figure 3:**
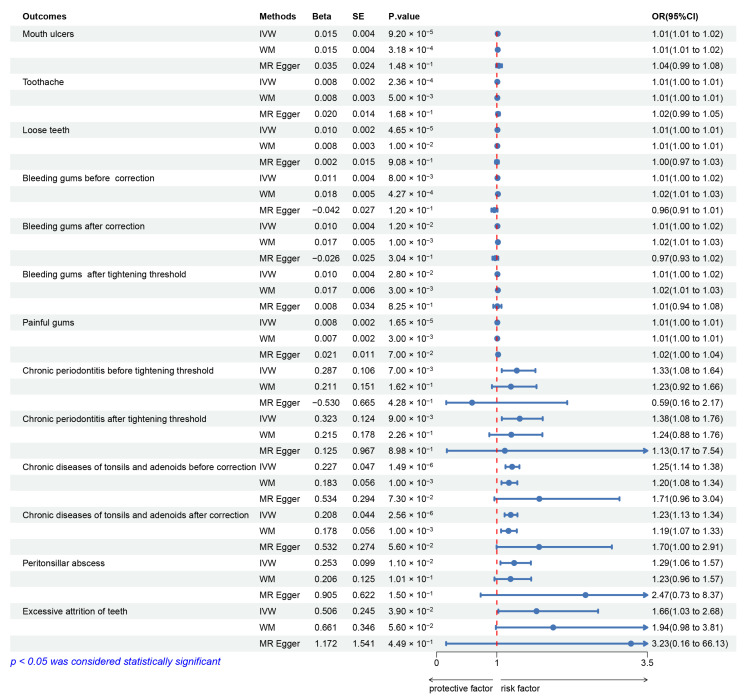
MR analysis from instrument variants for depression on the risk of 9 of the 17 studied oral diseases.

Genetic susceptibility to depression was positively correlated with 9 of the 17 oral diseases (mouth ulcers, toothache, loose teeth, bleeding gums, painful gums, chronic periodontitis, chronic tonsil and adenoid diseases, peritonsillar abscess, and excessive tooth attrition; *p* < 0.05 using the IVW method). These correlations persisted even after correcting multiple comparisons (Figure 3 and Table S3).

An increased possibility of developing mouth ulcers was observed to be associated with a genetic predisposition to depression (OR, 1.015; 95% CI: 1.007–1.022; *p* = 9.20 × 10^−5^), toothache (OR, 1.008; 95% CI: 1.004–1.013; *p* = 2.36 × 10^−4^), loose teeth (OR, 1.010; 95% CI: 1.005–1.014; *p* = 4.65 × 10^−5^), bleeding gums (OR, 1.011; 95% CI: 1.003–1.020; *p* = 0.008; without outliers: OR, 1.010; 95% CI: 1.002–1.018; *p* = 0.012), painful gums (OR, 1.008; 95% CI: 1.004–1.011; *p* = 1.65 × 10^−5^), chronic periodontitis (OR, 1.332; 95% CI: 1.082–1.641; *p* = 0.007), chronic tonsil and adenoid diseases (OR, 1.254; 95% CI: 1.144–1.375; *p* = 1.49 × 10^−6^; without outliers: OR, 1.231; 95% CI: 1.129–1.342; *p* = 2.56 × 10^−6^), peritonsillar abscess (OR, 1.288; 95% CI: 1.060–1.566; *p* = 0.011), and excessive tooth attrition (OR, 1.659; 95% CI: 1.026–2.682; *p* = 0.039). By using MR-PRESSO to eliminate the abnormal SNPs, a corrected effect estimate that demonstrated comparable outcomes was obtained.

The instrument *p*-value threshold was revised to 5 × 10^−9^ because the MR-Egger estimation of the MR analysis of bleeding gums and chronic periodontitis was inconsistent with the WM and IVW estimations [28]. We observed that depression was correlated with bleeding gums (OR, 1.010; 95% CI: 1.002–1.018; *p* = 0.012) and chronic periodontitis (OR, 1.381; 95% CI: 1.083–1.762; *p* = 0.009) either before or after tightening the instrument *p*-value threshold (Figure 3 and Table S3).

Furthermore, the scatter plot (Figures S1–S9) illustrated that patients with depression exhibited an increased susceptibility to mouth ulcers, toothache, loose teeth, bleeding gums, chronic tonsil and adenoid diseases, peritonsillar abscess, painful gums, chronic periodontitis, and excessive tooth attrition. In addition, Cochran’s Q statistic (MR-IVW) and Rucker’s Q statistic (MR-Egger) revealed the absence of heterogeneity in the MR analysis of depression and painful gums, chronic periodontitis, and excessive tooth attrition (*p* > 0.05); however, heterogeneity was observed in the MR analysis of depression and mouth ulcers, toothache, loose teeth, bleeding gums, chronic tonsil and adenoid diseases, and peritonsillar abscess (*p* < 0.05) (Table S4). Moreover, the MR-Egger intercept test revealed the absence of horizontal pleiotropy in the MR analyses of depression and mouth ulcers, toothache, loose teeth, bleeding gums, chronic tonsil and adenoid diseases, peritonsillar abscess, painful gums, chronic periodontitis, and excessive tooth attrition (*p* > 0.05) (Table S5). The leave-one-out method was used to analyze sensitivity (Figures S9–S18). The causality estimation conclusion for depression on mouth ulcers, toothache, loose teeth, bleeding gums, chronic tonsil and adenoid diseases, peritonsillar abscess, painful gums, chronic periodontitis, and excessive tooth attrition remained consistent and dependable even after removing any of the selected SNPs. As demonstrated in Figures S19–S27, the funnel plot revealed an approximate symmetry, suggesting the absence of directional pleiotropy. Collectively, our findings indicate their dependability.

**Figure 4:**
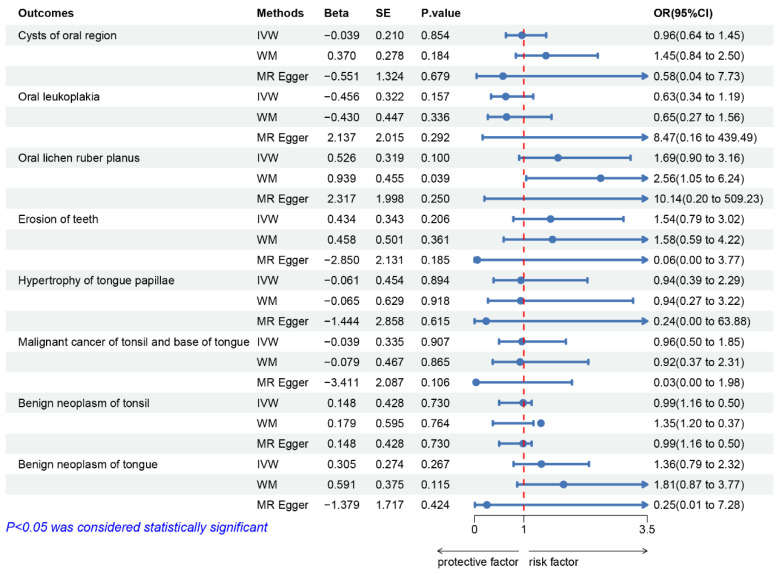
MR analysis from instrument variants for depression on the risk of 8 of the 17 studied oral diseases.

Using the three MR methods, no causal relationship was observed between depression and the remaining 8 of the 17 studied oral diseases (cysts of the oral region, oral leukoplakia, oral lichen ruber planus, dental erosion, hypertrophy of the tongue papillae, malignant cancer of the tonsil and base of the tongue, benign neoplasm of the tonsil, and benign neoplasm of the tongue; *p* > 0.05 using the IVW method) (Figure 4 and Table S6).

Cochran’s Q statistic (MR-IVW) and Rucker’s Q statistic (MR-Egger) revealed the absence of heterogeneity in the MR analyses of the following conditions: depression and cysts of the oral region, oral leukoplakia, oral lichen ruber planus, dental erosion, hypertrophy of the tongue papillae, malignant cancer of the tonsil and base of the tongue, benign neoplasm of the tonsil, and benign neoplasm of the tongue (*p* > 0.05) (Table S7). Furthermore, the MR-Egger intercept test revealed that the MR analysis of depression and cysts of the oral region, oral leukoplakia, oral lichen ruber planus, dental erosion, hypertrophy of the tongue papillae, malignant cancer of the tonsil and base of the tongue, benign neoplasm of the tonsil, and benign neoplasm of the tongue exhibited no horizontal pleiotropy (*p* > 0.05) (Table S8).

## 5. Discussion

In this study, by using publicly available GWAS summary statistics, we systematically performed MR analyses to elucidate the potential causal relationships between depression and 17 oral diseases. Our study findings suggest a potential association between genetic susceptibility to depression and an increased possibility of developing mouth ulcers, toothache, loose teeth, bleeding gums, painful gums, chronic periodontitis, chronic tonsil and adenoid diseases, peritonsillar abscess, and excessive tooth attrition. However, the evidence was insufficient to corroborate the correlation between depression and cysts of the oral region, oral leukoplakia, oral lichen ruber planus, dental erosion, hypertrophy of the tongue papillae, malignant cancer of the tonsil and base of the tongue, benign neoplasm of the tonsil, and benign neoplasm of the tongue.

In this study, a suggestive causal association was observed between depression and an increased risk of mouth ulcers. This observation corresponds to the outcomes of previous studies. The most prevalent ulcerative condition of the buccal cavity is mouth ulcers. Many studies have revealed that physiological disturbances because of emotions such as anxiety, wrath, mourning, or a sense of loss may contribute to oral ulcer development [49]. Furthermore, grief is associated with immune dysregulation, increasing the susceptibility to health issues associated with inflammation, including oral ulcers [50,51,52]. Our finding that a causal association exists between depression and the risk of toothache concurs with those of the Korean National Health and Nutrition Survey; in this survey involving participants who were diagnosed as having no toothache by a dentist, the self-reported prevalence of toothache was significantly higher in participants with depression than in those without depression [53]. In a previous study, a correlation was established between depression and elevated levels of proinflammatory cytokines, including interleukins and tumor necrosis factor, as well as increased expression of inflammatory molecules, as measured via acute phase proteins, including the C-reactive protein [54]. These cytokines mediate the peripheral sensitization of dental pain [55]. Bleeding gums, loose teeth, and painful gums are all prevalent oral diseases. Overall, our findings suggest causality between depression and bleeding gums, loose teeth, painful gums, and excessive tooth attrition. Recently, increasing evidence suggests that depression increases the incidence of bleeding gums, loose teeth, and painful gums. In a cross-sectional study involving 388 Portuguese students, a correlation was observed between perceived toothache and gingival hemorrhage and anxiety and depression. However, decreased dental care may not account for this association [56]. Furthermore, in a recent nationally representative prospective cohort study involving youth and adults in the United States, a consistent and stepwise increase in the prevalence of oral conditions, including bleeding gums, loose teeth, and gum disease, was observed at more severe levels of mental health issues [57]. Some studies have revealed that depression is correlated with an increased risk of awake and sleep bruxism; this in turn leads to excessive tooth attrition [58,59]. As the most prevalent form of periodontitis, comprising approximately 95% of patients, chronic periodontitis is brought about by the progression of chronic gingivitis into the deeper periodontal tissues. We observed that depression is a risk factor for chronic periodontitis. This finding is consistent with that of a previous study analyzing data from the US National Health and Nutrition Examination Survey [59]. Existing evidence indicates a delicate equilibrium between the host’s immune system and periodontal microbial flora [60]. Furthermore, increasing evidence suggests that the factors associated with depression, including dysregulated neurobiological and behavioral aspects, and an imbalance in the periodontal immune–microbiome, may significantly interact and thereby contribute to chronic periodontitis development and progression [61]. A peritonsillar abscess is an inflammation of the interstitial space surrounding the tonsils that is purulent in nature. Chronic tonsil and adenoid diseases are prevalent clinical oral diseases. In the present study, we observed that depression is a risk factor for peritonsillar abscess and chronic tonsil and adenoid diseases. At present, it is widely accepted that patients with depression experience innate and adaptive immune system dysregulation and that the dysregulation of their actions may contribute to tonsil and adenoid diseases [62,63]. Simultaneously, an imbalance in the homeostatic nature of the commensal–host relationship in the oropharynx, owing to compromised immunity, results in peritonsillar abscess [64].

Our study findings suggest the absence of causality between depression and cysts of the oral region, oral leukoplakia, oral lichen ruber planus, dental erosion, hypertrophy of the tongue papillae, malignant cancer of the tonsil and base of the tongue, benign neoplasm of the tonsil, and benign neoplasm of the tongue. Similarly, a previous cross-sectional study revealed that depression does not play a role in oral lichen planus development [65]. Although our findings indicate no causal relationship between depression and the incidence of malignant or benign tonsillar and tongue neoplasms, depression may affect the progression of such neoplasms. A study has revealed that depression inhibits natural killer cells and DNA repair enzymes, which are essential for defense mechanisms against cancer [66]. Furthermore, cortisol secretion is increased in patients with depression, and local cortisol production in the oral mucosa upregulates serum glucocorticoid-regulated kinase-1 (SGK-1) expression [67,68]. SGK-1 activation and expression can favor the invasiveness and metastasis of human tumors, including tongue cancer [69]. Simultaneously, some studies have revealed that patients with oral cancer and depression have higher mortality and recurrence rates than those without depression [70,71]. Therefore, considering the patient’s emotional regulation when preventing and treating related oral diseases is vital.

The primary strength of the present study was the MR study design, which minimized residual confounding and reversed causality, which are inherently observed in observational studies, and helped us investigate the potential causality between depression and oral diseases. The sample sizes of the IVs included in this study on depression were substantial, with the IVs demonstrating a robust association with focal exposure. Subsequently, this mitigated the effect of weak instrument bias and enhanced the statistical power of the study. Furthermore, we used the PhenoScanner database and individually examined the acquired SNPs associated with depression. The SNPs associated with potential confounders such as smoking, alcohol consumption, sugar consumption, wearing dentures, and vitamin A, B1, B2, C, D, and E deficiencies were removed. Fourth, we identified and eliminated outlier variants with horizontal pleiotropy using the MR-PRESSO test. Finally, the uniformity in sensitivity provided additional proof that the effect estimations are valid.

However, this study has some limitations that should be acknowledged. First, the results cannot be immediately generalized to other ethnic groups with different traditions and lifestyles because the participants in the datasets were of European ancestry. Second, because summary statistics were used and individual raw measurements were lacking, sex- or age-specific analysis could not be conducted; we will complement this section of the MR study when individual raw measurements are available in the database [72]. Third, multiple potential causal mechanisms may exist between depression and the nine oral diseases. While this constraint impedes us from conclusively establishing a particular causal pathway, our study findings can still be construed as indicating a causal association between depression and nine oral diseases, without identifying a specific pathway [73]. Because causation is inferred from genetics in MR analysis, it can only provide the potential causal linkages, but cannot identify the specific biological pathway that is responsible for this causality. Apart from the foregoing, other potentially influencing factors that are causing deviation may be present in our study, necessitating larger-scale MR analyses.

## 6. Conclusions

Our study results suggest a causal relationship between depression and a higher risk of oral diseases, namely, mouth ulcers, toothache, loose teeth, bleeding gums, painful gums, chronic periodontitis, chronic tonsil and adenoid diseases, peritonsillar abscess, and excessive tooth attrition. Our study provides new insights into the potential mechanism underlying the prediction of the occurrence and progression of oral diseases.

## Data Availability

The datasets analyzed in this study are summaries of publicly accessible statistics. Table S1 summarizes the information and data used to derive summary statistics. The datasets used/analyzed in this study are present in the Supplementary Materials.

## Supplementary Materials

The following supporting information can be downloaded at: https://www.mdpi.com/article/10.3390/genes14122191/s1. Table S1. Detailed information on depression and oral diseases; Table S2. Detailed data for selected gene instrumental variables in depression; Table S3. MR estimates for the association between depression and 9 (mouth ulcers, toothache, loose teeth, bleeding gums, painful gums, chronic periodontitis, chronic diseases of tonsils and adenoids, peritonsillar abscess, excessive attrition of teeth) of the 17 oral diseases; Table S4. Results of heterogeneity test between depression and 9 (mouth ulcers, toothache, loose teeth, bleeding gums, painful gums, chronic periodontitis, chronic diseases of tonsils and adenoids, peritonsillar abscess, excessive attrition of teeth) of the 17 studied oral diseases; Table S5. Results of MR-Egger intercept test between depression and 9 (mouth ulcers, toothache, loose teeth, bleeding gums, painful gums, chronic periodontitis, chronic diseases of tonsils and adenoids, peritonsillar abscess, excessive attrition of teeth) of the 17 studied oral diseases; Table S6. MR estimates for the association between depression and 8 (cysts of oral region, oral leukoplakia, oral lichen ruber planus, erosion of teeth, hypertrophy of tongue papillae, malignant cancer of tonsil and base of tongue, benign neoplasm of tonsil, benign neoplasm of tongue) of the 17 studied oral diseases; Table S7. Results of heterogeneity test between depression and 8 (cysts of oral region, oral leukoplakia, oral lichen ruber planus, erosion of teeth, hypertrophy of tongue papillae, malignant cancer of tonsil and base of tongue, benign neoplasm of tonsil, benign neoplasm of tongue) of the 17 studied oral; Table S8. Results of MR-Egger intercept test between depression and 8 (cysts of oral region, oral leukoplakia, oral lichen ruber planus, erosion of teeth, hypertrophy of tongue papillae, malignant cancer of tonsil and base of tongue, benign neoplasm of tonsil, benign neoplasm of tongue) of the 17 studied oral diseases; Figure S1. Scatter plots for the causal association between depression and mouth ulcers; Figure S2. Scatter plots for the causal association between depression and toothache; Figure S3. Scatter plots for the causal association between depression and loose teeth; Figure S4. Scatter plots for the causal association between depression and bleeding gums; Figure S5. Scatter plots for the causal association between depression and painful gums; Figure S6. Scatter plots for the causal association between depression and chronic periodontitis; Figure S7. Scatter plots for the causal association between depression and chronic diseases of tonsils and adenoids; Figure S8. Scatter plots for the causal association between depression and peritonsillar abscess; Figure S9. Scatter plots for the causal association between depression and excessive attrition of teeth; Figure S10. Leave-one-out plots for the causal association between depression and mouth ulcers; Figure S11. Leave-one-out plots for the causal association between depression and toothache; Figure S12. Leave-one-out plots for the causal association between depression and loose teeth; Figure S13. Leave-one-out plots for the causal association between depression and bleeding gums; Figure S14. Leave-one-out plots for the causal association between depression and painful gums; Figure S15. Leave-one-out plots for the causal association between depression and chronic periodontitis; Figure S16. Leave-one-out plots for the causal association between depression and chronic diseases of tonsils and adenoids; Figure S17. Leave-one-out plots for the causal association between depression and peritonsillar abscess; Figure S18. Leave-one-out plots for the causal association between depression and excessive attrition of teeth; Figure S19. Funnel plot for the causal association between depression and mouth ulcers; Figure S20. Funnel plot for the causal association between depression and toothache; Figure S21. Funnel plot for the causal association between depression and loose teeth; Figure S22. Funnel plot for the causal association between depression and bleeding gums; Figure S23. Funnel plot for the causal association between depression and painful gums; Figure S24. Funnel plot for the causal association between depression and chronic periodontitis; Figure S25. Funnel plot for the causal association between depression and chronic diseases of tonsils and adenoids; Figure S26. Funnel plot for the causal association between depression and peritonsillar abscess; Figure S27. Funnel plot for the causal association between depression and excessive attrition of teeth.
