# Supplementary material for: Potential Causal Association between Depression and Oral Diseases: A Mendelian Randomization Study
Source: Genes (Basel). 2023 Dec 8;14(12):2191. doi: 10.3390/genes14122191 (PMC10742945; doi:10.3390/genes14122191)
Supplement: Supplementary file 1 [file genes-14-02191-s001.zip › genes-2731740-supplementary.pdf]

Supporting information

Potential Causal Association Between Depression and Oral Diseases: A Mendelian Randomization Study

Supplementary Table S1. Detailed information on depression and oral diseases.

Supplementary Table S2. Detailed data for selected gene instrumental variables in depression.

Supplementary Table S3. MR estimates for the association between depression and 9 (mouth ulcers, toothache, loose teeth, bleeding gums, painful gums, chronic periodontitis, chronic diseases of tonsils and adenoids, peritonsillar abscess, excessive attrition of teeth) of the 17 oral diseases.

Supplementary Table S4. Results of heterogeneity test between depression and 9 (mouth ulcers, toothache, loose teeth, bleeding gums, painful gums, chronic periodontitis, chronic diseases of tonsils and adenoids, peritonsillar abscess, excessive attrition of teeth) of the 17 studied oral diseases

Supplementary Table S5. Results of MR-Egger intercept test between depression and 9 (mouth ulcers, toothache, loose teeth, bleeding gums, painful gums, chronic periodontitis, chronic diseases of tonsils and adenoids, peritonsillar abscess, excessive attrition of teeth) of the 17 studied oral diseases

Supplementary Table S6. MR estimates for the association between depression and 8 (cysts of oral region, oral leukoplakia, oral lichen ruber planus, erosion of teeth, hypertrophy of tongue papillae, malignant cancer of tonsil and base of tongue, benign neoplasm of tonsil, benign neoplasm of tongue) of the 17 studied oral diseases.

Supplementary Table S7. Results of heterogeneity test between depression and 8 (cysts of oral region, oral leukoplakia, oral lichen ruber planus, erosion of teeth, hypertrophy of tongue papillae, malignant cancer of tonsil and base of tongue, benign neoplasm of tonsil, benign neoplasm of tongue) of the 17 studied oral.

Supplementary Table S8. Results of MR-Egger intercept test between depression and 8 (cysts of oral region, oral leukoplakia, oral lichen ruber planus, erosion of teeth, hypertrophy of tongue papillae, malignant cancer of tonsil and base of tongue, benign neoplasm of tonsil, benign neoplasm of tongue) of the 17 studied oral diseases.

Supplementary Figure S1. Scatter plots for the causal association between depression and mouth ulcers.
Supplementary Figure S2. Scatter plots for the causal association between depression and toothache.
Supplementary Figure S3. Scatter plots for the causal association between depression and loose teeth.
Supplementary Figure S4. Scatter plots for the causal association between depression and bleeding gums.
Supplementary Figure S5. Scatter plots for the causal association between depression and painful gums.
Supplementary Figure S6. Scatter plots for the causal association between depression and chronic periodontitis.
Supplementary Figure S7. Scatter plots for the causal association between depression and chronic diseases of tonsils and adenoids.
Supplementary Figure S8. Scatter plots for the causal association between depression and peritonsillar abscess.
Supplementary Figure S9. Scatter plots for the causal association between depression and excessive attrition of teeth.
Supplementary Figure S10. Leave-one-out plots for the causal association between depression and mouth ulcers.
Supplementary Figure S11. Leave-one-out plots for the causal association between depression and toothache.
Supplementary Figure S12. Leave-one-out plots for the causal association between depression and loose teeth.
Supplementary Figure S13. Leave-one-out plots for the causal association between depression and bleeding gums.
Supplementary Figure S14. Leave-one-out plots for the causal association between depression and painful gums.
Supplementary Figure S15. Leave-one-out plots for the causal association between depression and chronic periodontitis.
Supplementary Figure S16. Leave-one-out plots for the causal association between depression and chronic diseases of tonsils and adenoids.
Supplementary Figure S17. Leave-one-out plots for the causal association between depression and peritonsillar abscess.
Supplementary Figure S18. Leave-one-out plots for the causal association between depression and excessive attrition of teeth.
Supplementary Figure S19. Funnel plot for the causal association between depression and mouth ulcers.
Supplementary Figure S20. Funnel plot for the causal association between depression and toothache.
Supplementary Figure S21. Funnel plot for the causal association between depression and loose teeth.
Supplementary Figure S22. Funnel plot for the causal association between depression and bleeding gums.

Supplementary Figure S23. Funnel plot for the causal association between depression and painful gums.
Supplementary Figure S24. Funnel plot for the causal association between depression and chronic periodontitis.
Supplementary Figure S25. Funnel plot for the causal association between depression and chronic diseases of tonsils and adenoids.
Supplementary Figure S26. Funnel plot for the causal association between depression and peritonsillar abscess.
Supplementary Figure S27. Funnel plot for the causal association between depression and excessive attrition of teeth.

Supplementary Table S1. Detailed information on depression and oral diseases.

**Attribute**	**Trait**	**Participants included in analysis**	**Age**	**Sex**	**Consortium**	**GWAS ID/PubMed ID**
Exposure	Depression	246,363 cases and 561,190 controls of European ancestry	Adults	Males and Females	the Psychiatric Genomics Consortium, UK Biobank study, and 23andMe	30718901
Outcome	Mouth ulcers	47,102cases and 414,011 controls of European ancestry	NA	Males and Females	UK Biobank	ukb-b-6458
Outcome	Cysts of oral region	122,3 cases and 259,234 controls of European ancestry	NA	Males and Females	FinnGen	finngen_R9_K11_ORALCYST
Outcome	Oral leukoplakia	474 cases and 376,803 controls of European ancestry	NA	Males and Females	FinnGen	finngen_R9_K11_ORAL_LEUCOPLACIA
Outcome	Oral lichen ruber planus	510 cases and 376,767controls of European ancestry	NA	Males and Females	FinnGen	finngen_R9_K11_ORAL_LICHEN_PLANUS
Outcome	Toothache	18,964 cases and 442,149 controls of European ancestry	NA	Males and Females	UK Biobank	ukb-b-19191
Outcome	Loose teeth	18,981 cases and 442,132 controls of European ancestry	NA	Males and Females	UK Biobank	ukb-b-12849
Outcome	Bleeding gums	60,218 cases and 400,895 controls of European ancestry	NA	Males and Females	UK Biobank	ukb-b-7872
Outcome	Painful gums	13,314 cases and 447,799 controls of European ancestry	NA	Males and Females	UK Biobank	ukb-b-11161
Outcome	Chronic periodontitis	4,434 cases and 259,234 controls of European ancestry	NA	Males and Females	FinnGen	finngen_R9_K11_PERIODON_CHRON
Outcome	Excessive attrition of teeth	840 cases and 259,234 controls of European ancestry	NA	Males and Females	FinnGen	finngen_R9_K11_ATTRITION
Outcome	Erosion of teeth	425 cases and 259,234 controls of European ancestry	NA	Males and Females	FinnGen	finngen_R9_K11_EROSION
Outcome	Chronic diseases of tonsils and adenoids	43,325 cases and 283,342 controls of European ancestry	NA	Males and Females	FinnGen	finngen_R9_J10_CHRONTONSADEN
Outcome	Peritonsillar abscess	7,510 cases and 283,342 controls of European ancestry	NA	Males and Females	FinnGen	finngen_R9_J10_PERITONSABSC

Outcome	Benign neoplasm: Tonsil	281 cases and 376,996 controls of European ancestry	NA	Males and Females	FinnGen	finngen_R9_CD2_BENIGN_TONSIL
Outcome	Malignant cancer of tonsil and base of tongue	443 cases and 287,137 controls of European ancestry	NA	Males and Females	FinnGen	finngen_R9_C3_TONSIL_BASETONGUE
Outcome	Hypertrophy of tongue papillae	268 cases and 377,009 controls of European ancestry	NA	Males and Females	FinnGen	finngen_R9_K11_HYPERTROPHY_TONGUE_PAPILLAE
Outcome	Benign neoplasm: Tongue	720 cases and 376,557 controls of European ancestry	NA	Males and Females	FinnGen	finngen_R9_CD2_BENIGN_TONGUE

Abbreviations: NA, not available.

Supplementary Table S2. Detailed data for selected gene instrumental variables in depression.

SNP	Gene	effect_allele	other_allele	EAF	Beta	SE	*P*-value	N	R2	F-statistic
rs1002656	RP4-614N24.1	T	C	0.7033	-0.0266	0.0038	3.74E-12	807,553	6.07E-05	48.99988
rs10789214	SGIP1	T	C	0.5661	0.0193	0.0035	4.44E-08	807,553	3.77E-05	30.40727
rs10913112	RFWD2	T	C	0.3767	-0.0264	0.0036	3.40E-13	807,553	6.66E-05	53.77764
rs113188507	AL606519.1	A	G	0.2838	0.0221	0.0039	1.87E-08	807,553	3.98E-05	32.11103
rs11579246	ELAVL4	A	G	0.9067	0.0381	0.0061	5.71E-10	807,553	4.83E-05	39.01119
rs1466887	RP5-1180C18.1	T	C	0.5511	-0.0199	0.0036	4.12E-08	807,553	3.78E-05	30.55625
rs169235	CACNA1E	A	G	0.753	-0.0229	0.0041	2.98E-08	807,553	3.86E-05	31.19623
rs1890946	NRDC	T	C	0.4671	-0.0235	0.0035	2.68E-11	807,553	5.58E-05	45.08152
rs2568958	RPL31P12	A	G	0.6156	0.0373	0.0036	8.47E-25	807,553	1.33E-04	107.3524
rs301799	RERE	T	C	0.5694	-0.025	0.0035	1.36E-12	807,553	6.32E-05	51.02028
rs72710803	RP1-35C21.2	A	C	0.9121	-0.041	0.0062	5.29E-11	807,553	5.41E-05	43.73038
rs1226412	LINC01876	T	C	0.7917	0.0256	0.0043	3.46E-09	807,553	4.39E-05	35.44394
rs1568452	ACTG1P22	T	C	0.3851	0.0248	0.0036	8.12E-12	807,553	5.88E-05	47.45667
rs62188629	AC007879.1	A	G	0.3136	0.0236	0.0038	7.13E-10	807,553	4.78E-05	38.57054
rs7585722	RNF103-CHMP3	T	C	0.8458	-0.0269	0.0048	2.68E-08	807,553	3.89E-05	31.40661
rs1095626	RSRC1	T	C	0.5799	-0.0264	0.0035	7.13E-14	807,553	7.04E-05	56.89455
rs13084037	KLHDC8B	A	G	0.774	-0.0245	0.0042	7.08E-09	807,553	4.21E-05	34.02769
rs141954845	FHIT	A	G	0.388	0.0229	0.0037	8.15E-10	807,553	4.74E-05	38.30597
rs4346585	RP11-944L7.4	T	C	0.696	-0.0236	0.0038	7.13E-10	807,553	4.78E-05	38.57054
rs6783233	RP11-384F7.2	T	C	0.2833	0.0218	0.0039	2.90E-08	807,553	3.87E-05	31.24516
rs7624336	AC097015.1	T	G	0.2087	0.0238	0.0043	3.96E-08	807,553	3.79E-05	30.63486
rs34937911	BEND4	T	C	0.8838	0.0304	0.0055	4.13E-08	807,553	3.78E-05	30.55067
rs35553410	RP11-404I7.1	T	C	0.7462	-0.0244	0.004	1.42E-09	807,553	4.61E-05	37.20991
rs45510091	KIAA1109	A	G	0.9472	0.0448	0.008	1.83E-08	807,553	3.88E-05	31.35992
rs7659414	RN7SKP13	A	C	0.5782	-0.0201	0.0035	1.20E-08	807,553	4.08E-05	32.98033
rs7685686	HTT	A	G	0.5753	0.0202	0.0036	2.57E-08	807,553	3.90E-05	31.48449
rs10061069	POU5F2	C	G	0.2212	-0.0275	0.0042	8.15E-11	807,553	5.31E-05	42.87121
rs11135349	CTC-340A15.2	A	C	0.4713	-0.0295	0.0035	6.04E-17	807,553	8.80E-05	71.04064
rs30266	RP11-6N13.1	A	G	0.3296	0.0308	0.0037	1.45E-16	807,553	8.58E-05	69.2942
rs3099439	TMEM161B	T	C	0.5288	-0.0276	0.0035	5.05E-15	807,553	7.70E-05	62.18434

rs12052908	--	A	T	0.5325	-0.022	0.0035	4.44E-10	807,553	4.89E-05	39.51011
rs60157091	AC010376.1	T	C	0.515	0.02	0.0035	1.42E-08	807,553	4.04E-05	32.65298
rs1933802	LIN28B-AS1	C	G	0.4536	-0.0223	0.0035	2.57E-10	807,553	5.03E-05	40.595
rs2029865	XX-C2158C12.1	A	T	0.4534	-0.0201	0.0035	1.20E-08	807,553	4.08E-05	32.98033
rs2876520	AL356739.1	C	G	0.5271	-0.023	0.0036	2.29E-10	807,553	5.05E-05	40.8178
rs725616	SAMD5	T	C	0.3644	0.0204	0.0036	1.87E-08	807,553	3.98E-05	32.11103
rs7758630	RP3-359N14.1	A	T	0.4051	-0.0225	0.0036	5.56E-10	807,553	4.84E-05	39.0624
rs9363467	RNU7-66P	T	C	0.6035	0.0237	0.0036	6.44E-11	807,553	5.37E-05	43.34017
rs16887442	AC079799.2	T	C	0.4347	0.0203	0.0035	8.62E-09	807,553	4.17E-05	33.63992
rs2043539	TMEM106B	A	G	0.4177	0.0273	0.0035	9.89E-15	807,553	7.53E-05	60.83985
rs2247523	PCLO	C	G	0.5319	-0.0207	0.0035	4.38E-09	807,553	4.33E-05	34.97869
rs3823624	MAD1L1	T	C	0.8067	0.0272	0.0045	1.99E-09	807,553	4.52E-05	36.53522
rs58104186	AC073071.1	A	G	0.4689	0.0237	0.0035	1.82E-11	807,553	5.68E-05	45.85213
rs7807677	CTTNBP2	T	C	0.5505	0.0237	0.0035	1.82E-11	807,553	5.68E-05	45.85213
rs67436663	RP11-333A23.1	C	G	0.2402	-0.0259	0.0042	9.37E-10	807,553	4.71E-05	38.02768
rs7837935	CYP7B1	T	G	0.1522	-0.0292	0.0049	3.34E-09	807,553	4.40E-05	35.51178
rs10817969	ASTN2	T	G	0.7173	0.0261	0.0039	3.11E-11	807,553	5.55E-05	44.78687
rs1354115	CARM1P1	A	C	0.6243	0.021	0.0036	7.08E-09	807,553	4.21E-05	34.02769
rs1982277	RP11-32D4.1	T	C	0.7594	0.0279	0.0041	1.45E-11	807,553	5.73E-05	46.30625
rs263645	RP11-132E11.2	A	T	0.5438	0.0221	0.0035	3.70E-10	807,553	4.94E-05	39.87011
rs2670139	DENND1A	T	C	0.7609	-0.0266	0.0041	1.21E-10	807,553	5.21E-05	42.09151
rs3793577	ELAVL2	A	G	0.4665	-0.0229	0.0035	8.41E-11	807,553	5.30E-05	42.80887
rs59283172	RN7SKP120	A	G	0.1069	-0.0329	0.0057	1.02E-08	807,553	4.13E-05	33.31509
rs7030813	PAX5	T	C	0.3736	0.0253	0.0036	3.07E-12	807,553	6.12E-05	49.38954
rs913930	TLR4	A	G	0.6433	-0.0208	0.0037	2.42E-08	807,553	3.91E-05	31.60255
rs1021363	SORCS3	A	G	0.3547	0.0303	0.0037	4.41E-16	807,553	8.30E-05	67.06265
rs997934	ADARB2	T	C	0.3795	0.0198	0.0036	4.81E-08	807,553	3.75E-05	30.24993
rs1448938	DCDC1	A	G	0.4171	0.0214	0.0035	1.30E-09	807,553	4.63E-05	37.3844
rs198457	DAGLA	T	C	0.1925	-0.0292	0.0046	2.99E-10	807,553	4.99E-05	40.2948
rs2187490	Y_RNA	T	G	0.9106	-0.0338	0.0061	3.82E-08	807,553	3.80E-05	30.70242
rs2509805	RP11-734C14.2	T	C	0.3209	0.022	0.0038	9.17E-09	807,553	4.15E-05	33.51792
rs57344483	CTD-2234N14.2	A	G	0.9259	-0.038	0.0068	1.82E-08	807,553	3.87E-05	31.2283
rs58621819	LTBP3	A	T	0.7903	-0.0245	0.0043	1.57E-08	807,553	4.02E-05	32.46341
rs61902811	DRD2	A	G	0.3682	-0.0257	0.0036	1.40E-12	807,553	6.31E-05	50.96361
rs7117514	SHANK2	A	G	0.5417	-0.0204	0.0035	7.29E-09	807,553	4.21E-05	33.97216

rs7932640	GRM5	T	C	0.4417	0.0281	0.0035	1.62E-15	807,553	7.98E-05	64.4578
rs10774600	ATP2A2	T	C	0.1656	-0.0267	0.0048	3.39E-08	807,553	3.83E-05	30.94133
rs3213572	SPPL3	A	G	0.4745	0.0217	0.0035	7.61E-10	807,553	4.76E-05	38.4399
rs56314503	SNORA3	T	G	0.7487	-0.0254	0.004	2.95E-10	807,553	4.99E-05	40.3224
rs78337797	SOX5	T	G	0.8781	0.0306	0.0055	3.37E-08	807,553	3.83E-05	30.95397
rs1343605	OLFM4	A	C	0.384	0.0313	0.0036	6.23E-18	807,553	9.36E-05	75.59318
rs1409379	B3GLCT	T	C	0.7641	0.0249	0.0041	1.67E-09	807,553	4.57E-05	36.88331
rs4772087	STK24	T	C	0.3732	0.0227	0.0036	3.91E-10	807,553	4.92E-05	39.75993
rs9545360	SPRY2	A	C	0.1807	-0.0271	0.0046	5.02E-09	807,553	4.30E-05	34.70738
rs9592461	PCDH9	A	G	0.4874	0.0216	0.0035	9.10E-10	807,553	4.72E-05	38.08644
rs10149470	BAG5	A	G	0.4869	-0.0267	0.0035	3.72E-14	807,553	7.21E-05	58.19496
rs1045430	AREL1	T	G	0.4792	-0.0253	0.0035	7.31E-13	807,553	6.47E-05	52.25212
rs1152578	ESR2	T	C	0.4357	-0.0218	0.0035	6.36E-10	807,553	4.80E-05	38.79501
rs1956373	RTN1	T	G	0.7436	-0.0226	0.004	2.06E-08	807,553	3.95E-05	31.92242
rs61990288	LRFN5	A	G	0.5083	-0.026	0.0035	1.68E-13	807,553	6.83E-05	55.18354
rs34488670	SEMA6D	T	C	0.7887	-0.0252	0.0043	6.03E-09	807,553	4.25E-05	34.34497
rs8037355	RP11-720L8.1	T	C	0.5556	-0.0233	0.0035	3.94E-11	807,553	5.49E-05	44.31744
rs12923444	METTL9	A	C	0.5625	-0.0214	0.0035	1.30E-09	807,553	4.63E-05	37.3844
rs56887639	U95743.1	A	G	0.7264	-0.0278	0.0039	1.51E-12	807,553	6.29E-05	50.81118
rs7193263	RBFOX1	A	G	0.6679	-0.0239	0.0038	4.33E-10	807,553	4.90E-05	39.55738
rs7198928	RBFOX1	T	C	0.6159	0.0239	0.0036	4.45E-11	807,553	5.46E-05	44.07474
rs7200826	SHISA9	T	C	0.2551	0.028	0.004	3.74E-12	807,553	6.07E-05	48.99988
rs75581564	PIPOX	A	G	0.1165	0.0301	0.0054	3.17E-08	807,553	3.85E-05	31.07022
rs12967143	TCF4	C	G	0.6984	-0.0312	0.0038	3.70E-16	807,553	8.35E-05	67.41258
rs12967855	CELF4	A	G	0.3295	0.0265	0.0037	1.18E-12	807,553	6.35E-05	51.29644
rs7227069	DCC	A	G	0.4326	0.0238	0.0035	1.50E-11	807,553	5.73E-05	46.23989
rs7241572	RP11-154H12.3	A	G	0.201	0.028	0.0044	2.70E-10	807,553	5.01E-05	40.49577
rs33431	ZNF536	T	C	0.6144	0.0198	0.0036	4.81E-08	807,553	3.75E-05	30.24993
rs12624433	SLC12A5	A	G	0.2584	0.0233	0.004	7.44E-09	807,553	4.20E-05	33.93054
rs143186028	EMILIN3	T	G	0.1778	0.0277	0.0046	2.29E-09	807,553	4.49E-05	36.26125
rs5995992	EP300	T	C	0.7155	-0.0266	0.0039	1.30E-11	807,553	5.76E-05	46.51928

Supplementary Table S3. MR estimates for the association between depression and 9 (mouth ulcers, toothache, loose teeth, bleeding gums, painful gums, chr onic periodontitis, chronic diseases of tonsils and adenoids, peritonsillar abscess, excessive attrition of teeth) of the 17 oral diseases.

**Outcomes**	**Expose**	**Methods**	**Total cases**	**Total controls**	**SNP (n)**	**Beta**	**SE**	**P-value**	**Beta-low**	**Beta-up**	**OR**	**OR-low**	**OR-up**
Mouth ulcers	Depression	IVW	47,102	414,011	89	0.015	0.004	9.20 E-05	0.007	0.022	1.015	1.007	1.022
		MR Egger	47,102	414,011	89	0.035	0.024	1.48 E-01	-0.012	0.082	1.036	0.988	1.085
		WM	47,102	414,011	89	0.015	0.004	3.18 E-04	0.007	0.024	1.015	1.007	1.024
Toothache	Depression	IVW	18,964	442,149	89	0.008	0.002	2.36 E-04	0.004	0.013	1.008	1.004	1.013
		MR Egger	18,964	442,149	89	0.020	0.014	1.68 E-01	-0.008	0.048	1.020	0.992	1.049
		WM	18,964	442,149	89	0.008	0.003	4.55 E-03	0.003	0.014	1.008	1.003	1.014
Loose teeth	Depression	IVW	18,981	442,132	89	0.010	0.002	4.65 E-05	0.005	0.014	1.010	1.005	1.014
		MR Egger	18,981	442,132	89	0.002	0.015	9.08 E-01	-0.028	0.032	1.002	0.972	1.032
		WM	18,981	442,132	89	0.008	0.003	1.02 E-02	0.002	0.014	1.008	1.002	1.014
Bleeding gums befoe before correction	Depression	IVW	60,218	400,895	89	0.011	0.004	0.008	0.003	0.020	1.011	1.003	1.020
		MR Egger	60,218	400,895	89	-0.042	0.027	0.120	-0.095	0.010	0.959	0.910	1.011
		WM	60,218	400,895	89	0.018	0.005	0.000	0.008	0.027	1.018	1.008	1.028
Bleeding gums befoe after correction	Depression	IVW	60,218	400,895	88	0.010	0.004	0.012	0.002	0.018	1.010	1.002	1.018
		MR Egger	60,218	400,895	88	-0.026	0.025	0.304	-0.076	0.024	0.974	0.927	1.024
		WM	60,218	400,895	88	0.017	0.005	0.001	0.007	0.028	1.018	1.007	1.028
Bleeding gums befoe after tightening threshold	Depression	IVW	60,218	400,895	57	0.010	0.004	0.028	0.001	0.018	1.010	1.001	1.019
		MR Egger	60,218	400,895	57	0.008	0.034	0.825	-0.059	0.075	1.008	0.942	1.077
		WM	60,218	400,895	57	0.017	0.006	0.003	0.006	0.028	1.017	1.006	1.028
Painful gums	Depression	IVW	13,314	447,799	89	0.008	0.002	1.65 E-05	0.004	0.011	1.008	1.004	1.011

		MR Egger	13,314	447,799	89	0.021	0.011	6.99 E-02	-0.001	0.042	1.021	0.999	1.043
		WM	13,314	447,799	89	0.007	0.002	3.26 E-03	0.002	0.012	1.007	1.002	1.012
Chronic periodontitis	Depression	IVW	4,434	259,234	80	0.287	0.106	0.007	0.078	0.495	1.332	1.082	1.641
		MR Egger	4,434	259,234	80	-0.530	0.665	0.428	-1.834	0.774	0.589	0.160	2.169
		WM	4,434	259,234	80	0.211	0.151	0.162	-0.085	0.506	1.234	0.919	1.659
Chronic diseases of tonsils and adenoids	Depression	IVW	43,325	283,342	80	0.227	0.047	1.49 E-06	0.134	0.319	1.254	1.144	1.375
		MR Egger	43,325	283,342	80	0.534	0.294	7.32 E-02	-0.042	1.111	1.706	0.959	3.037
		WM	43,325	283,342	80	0.183	0.056	9.79 E-04	0.074	0.292	1.201	1.077	1.340
Peritonsillar abscess	Depression	IVW	7,510	283,342	80	0.253	0.099	0.011	0.058	0.448	1.288	1.060	1.566
		MR Egger	7,510	283,342	80	0.905	0.622	0.150	-0.314	2.125	2.473	0.731	8.369
		WM	7,510	283,342	80	0.206	0.125	0.101	-0.040	0.451	1.228	0.961	1.571
Excessive attrition of teeth	Depression	IVW	840	259,234	80	0.506	0.245	0.039	0.026	0.987	1.659	1.026	2.682
		MR Egger	840	259,234	80	1.172	1.541	0.449	-1.848	4.192	3.228	0.158	66.130
		WM	840	259,234	80	0.661	0.346	0.056	-0.017	1.338	1.936	0.984	3.812

Supplementary Table S4. Results of heterogeneity test between depression and 9 (mouth ulcers, toothache, loose teeth, bleeding gums, painful gums, chronic periodontitis, chronic diseases of tonsils and adenoids, peritonsillar abscess, excessive attrition of teeth) of the 17 studied oral diseases.

**Outcomes**	**Exposure**	**Cochran's Q Test**		**Rucker's Q Test**	
		**MR-egger**		**IVW**	
		**Q**	***P*-value**	**Q**	***P*-value**
Mouth ulcers	Depression	144.2548	1.12E-04	145.4618	1.14E-04
Toothache	Depression	118.5694	0.014	119.4874	0.014
Loose teeth	Depression	134.2261	8.71E-04	134.659	0.001
Bleeding gums before correction	Depression	146.094	7.54E-05	152.9461	2.20E-05
Bleeding gums after correction	Depression	126.1004	0.003	129.1611	0.002
Bleeding gums after tightening threshold	Depression	74.80412	0.039	74.80968	0.047
Painful gums	Depression	102.0261	0.129	103.6301	0.122
Chronic periodontitis before tightening threshold	Depression	58.05752	0.956	59.6029	0.949
Chronic periodontitis after tightening threshold	Depression	38.61956	0.856	38.66238	0.878
Chronic diseases of tonsils and adenoids before correction	Depression	124.1317	6.97E-04	125.9186	6.25E-04
Chronic diseases of tonsils and adenoids after correction	Depression	106.0661	0.016	108.0501	0.014
Peritonsillar abscess	Depression	113.796	0.005	115.4408	0.005
Excessive attrition of teeth	Depression	80.65823	0.396	80.85634	0.421

Supplementary Table S5. Results of MR-Egger intercept test between depression and 9 (mouth ulcers, toothache, loose teeth, bleeding gums, painful gums, chronic periodontitis, chronic diseases of tonsils and adenoids, peritonsillar abscess, excessive attrition of teeth) of the 17 studied oral diseases.

**Outcome**	**Exposure**	**Egger intercept**	**SE**	***P*-value**
Mouth ulcers	Depression	-0.001	0.001	0.396
Toothache	Depression	-2.97E-04	3.62E-04	0.414
Loose teeth	Depression	2.04E-04	3.85E-04	0.598
Bleeding gums before correction	Depression	0.001	0.001	0.046
Bleeding gums after correction	Depression	0.001	0.001	0.152
Bleeding gums after tightening threshold	Depression	5.72E-05	0.001	0.949
Painful gums	Depression	-3.31E-04	0.000	0.245
Chronic periodontitis before tightening threshold	Depression	0.021	0.017	0.218
Chronic periodontitis after tightening threshold	Depression	0.005	0.025	0.837
Chronic diseases of tonsils and adenoids before correction	Depression	-0.008	0.007	0.293
Chronic diseases of tonsils and adenoids after correction	Depression	-0.008	0.007	0.234
Peritonsillar abscess	Depression	-0.017	0.016	0.292
Excessive attrition of teeth	Depression	-0.017	0.039	0.663

Supplementary Table S6. MR estimates for the association between depression and 8 (cysts of oral region, oral leukoplakia, oral lichen ruber planus, erosion of teeth, hypertrophy of tongue papillae, malignant cancer of tonsil and base of tongue, benign neoplasm of tonsil, benign neoplasm of tongue) of the 17 studied oral diseases.

**Outcomes**	**Expose**	**Methods**	**Total cases**	**Total controls**	**SNP (n)**	**Beta**	**SE**	***P*-value**	**Beta-low**	**Beta-up**	**OR**	**OR-low**	**OR-up**
Cysts of oral region	Depression	IVW	1,223	259,234	80	-0.039	0.210	0.854	-0.451	0.374	0.962	0.637	1.453
		WM	1,223	259,234	80	0.370	0.278	0.184	-0.175	0.916	1.448	0.839	2.499
		MR Egger	1,223	259,234	80	-0.551	1.324	0.679	-3.146	2.045	0.577	0.043	7.726
Oral leukoplakia	Depression	IVW	474	376,803	80	-0.456	0.322	0.157	-1.087	0.175	0.634	0.337	1.192
		WM	474	376,803	80	-0.430	0.447	0.336	-1.307	0.446	0.650	0.271	1.562
		MR Egger	474	376,803	80	2.137	2.015	0.292	-1.812	6.086	8.473	0.163	439.490
Oral lichen ruber planus	Depression	IVW	510	376,767	80	0.526	0.319	0.100	-0.100	1.152	1.692	0.905	3.164
		WM	510	376,767	80	0.939	0.455	0.039	0.048	1.831	2.558	1.049	6.240
		MR Egger	510	376,767	80	2.317	1.998	0.250	-1.600	6.233	10.140	0.202	509.230
Erosion of teeth	Depression	IVW	425	259,234	80	0.434	0.343	0.206	-0.238	1.106	1.543	0.788	3.021
		WM	425	259,234	80	0.458	0.501	0.361	-0.524	1.441	1.581	0.592	4.224
		MR Egger	425	259,234	80	-2.850	2.131	0.185	-7.027	1.327	0.058	0.001	3.770
Hypertrophy of tongue papillae	Depression	IVW	268	377,009	80	-0.061	0.454	0.894	-0.950	0.829	0.941	0.387	2.290
		WM	268	377,009	80	-0.065	0.629	0.918	-1.298	1.168	0.937	0.273	3.216
		MR Egger	268	377,009	80	-1.444	2.858	0.615	-7.044	4.157	0.236	0.001	63.883
Malignant cancer of	Depression	IVW	443	287,137	80	-0.039	0.335	0.907	-0.695	0.617	0.962	0.499	1.854

tonsil and base of tongue		WM	443	287,137	80	-0.079	0.467	0.865	-0.995	0.837	0.924	0.370	2.308
		MR Egger	443	287,137	80	-3.411	2.087	0.106	-7.501	0.680	0.033	0.001	1.975
Benign neoplasm of tonsil	Depression	IVW	281	376,996	80	0.148	0.428	0.730	-0.691	-0.696	0.986	1.159	0.501
		WM	281	376,996	80	0.179	0.595	0.764	-0.988	-0.987	1.346	1.196	0.372
		MR Egger	281	376,996	80	0.148	0.428	0.730	-0.691	-0.696	0.986	1.159	0.501
Benign neoplasm of tongue	Depression	IVW	720	376,557	80	0.305	0.274	0.267	-0.233	0.842	1.356	0.792	2.321
		WM	720	376,557	80	0.591	0.375	0.115	-0.144	1.327	1.806	0.866	3.768
		MR Egger	720	376,557	80	-1.379	1.717	0.424	-4.744	1.986	0.252	0.009	7.283

Supplementary Table S7. Results of heterogeneity test between depression and 8 (cysts of oral region, oral leukoplakia, oral lichen ruber planus, erosion of teeth, hypertrophy of tongue papillae, malignant cancer of tonsil and base of tongue, benign neoplasm of tonsil, benign neoplasm of tongue) of the 17 studied oral diseases.

**Outcomes**	**Exposure**	**Cochran's Q Test**		**Rucker's Q Test**	
		**MR-egger**		**IVW**	
		**Q**	***P*-value**	**Q**	***P*-value**
Cysts of oral region	Depression	86.598	0.237	86.768	0.257
Oral leukoplakia	Depression	58.756	0.949	60.456	0.940
Oral lichen ruber planus	Depression	82.624	0.339	83.497	0.343
Erosion of teeth	Depression	77.699	0.488	80.135	0.443
Hypertrophy of tongue papillae	Depression	88.723	0.191	88.996	0.207
Malignant cancer of tonsil and base of tongue	Depression	77.029	0.510	79.706	0.457
Benign neoplasm of tonsil	Depression	82.088	0.354	82.851	0.362
Benign neoplasm of tongue	Depression	85.916	0.253	87.003	0.252

Supplementary Table S8: Results of MR-Egger intercept test between depression and 8 (cysts of oral region, oral leukoplakia, oral lichen ruber planus, erosion of teeth, hypertrophy of tongue papillae, malignant cancer of tonsil and base of tongue, benign neoplasm of tonsil, benign neoplasm of tongue) of the 17 studied oral diseases.

**Outcome**	**Exposure**	**Egger intercept**	**SE**	***P*-value**
Cysts of oral region	Depression	0.013	0.033	0.696
Oral leukoplakia	Depression	-6.63E-02	5.09E-02	0.196
Oral lichen ruber planus	Depression	-4.58E-02	5.05E-02	0.367
Erosion of teeth	Depression	0.084	0.054	0.123
Hypertrophy of tongue papillae	Depression	0.035	0.072	0.625
Malignant cancer of tonsil and base of tongue	Depression	8.62E-02	0.053	0.106
Benign neoplasm of tonsil	Depression	5.77E-02	0.068	0.397
Benign neoplasm of tongue	Depression	0.043	0.043	0.324

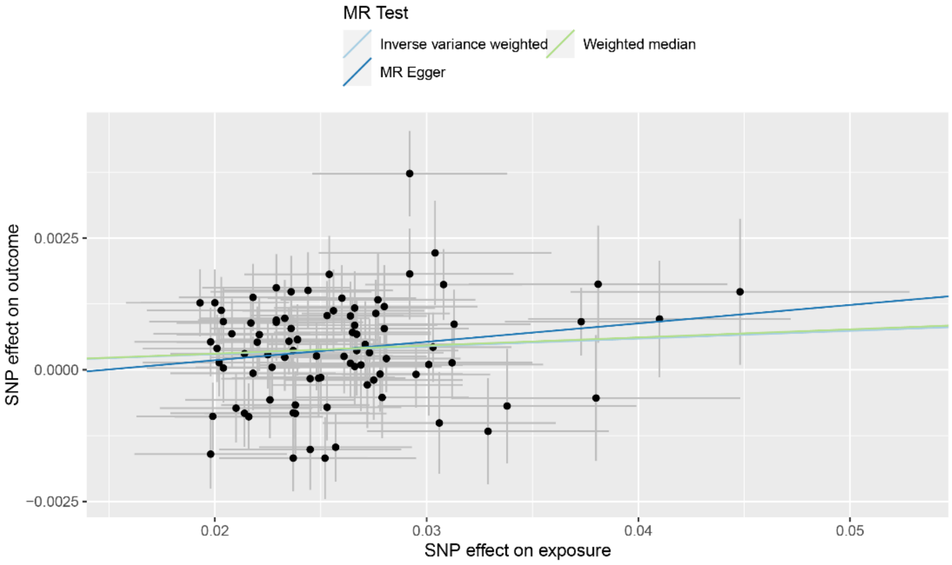

Supplementary Figure S1. Scatter plot for the causal association between depression and mouth ulcers

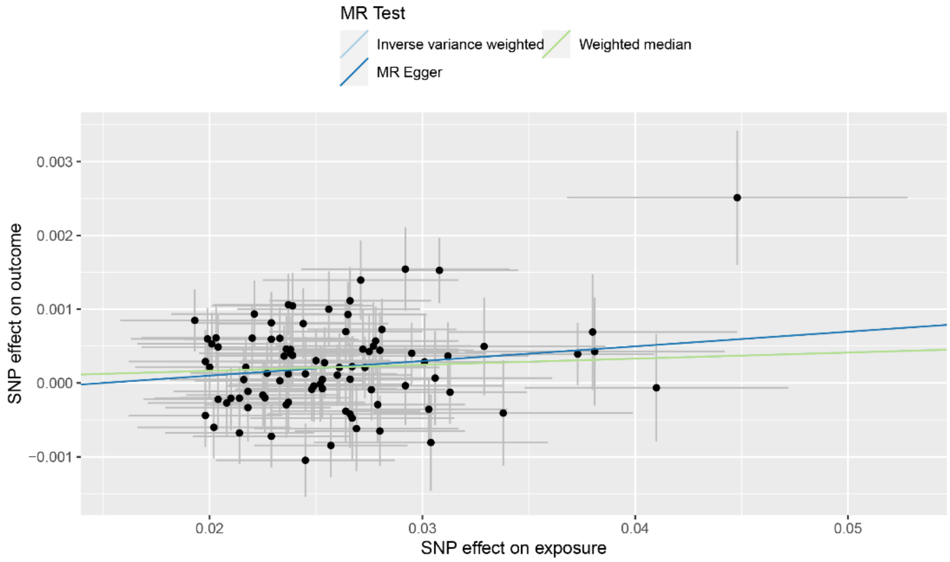

Supplementary Figure S2. Scatter plots for the causal association between depression and toothache.

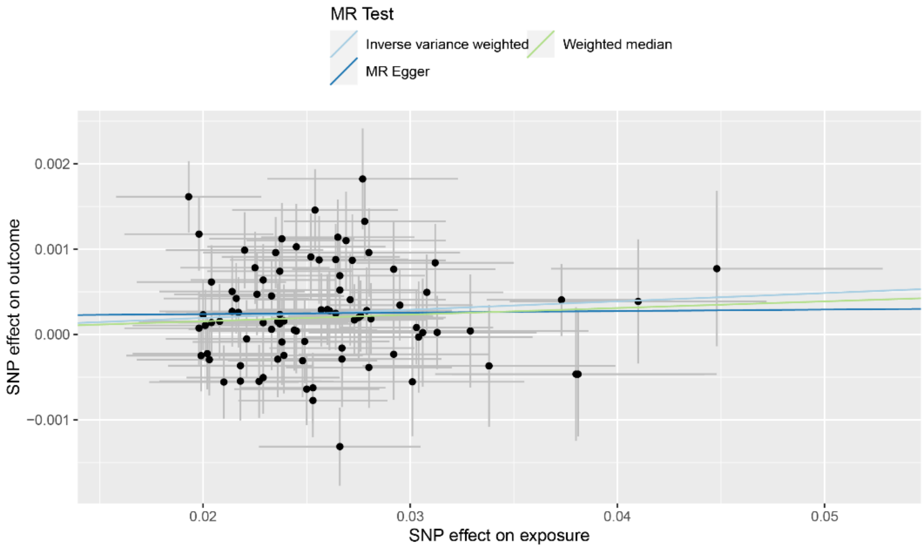

Supplementary Figure S3. Scatter plots for the causal association between depression and loose teeth.

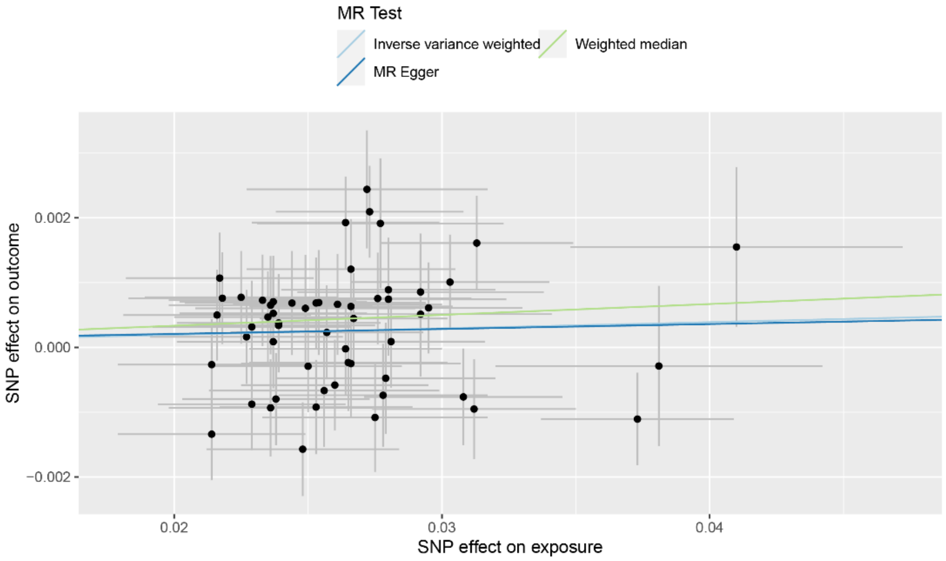

Supplementary Figure S4. Scatter plots for the causal association between depression and bleeding gums.

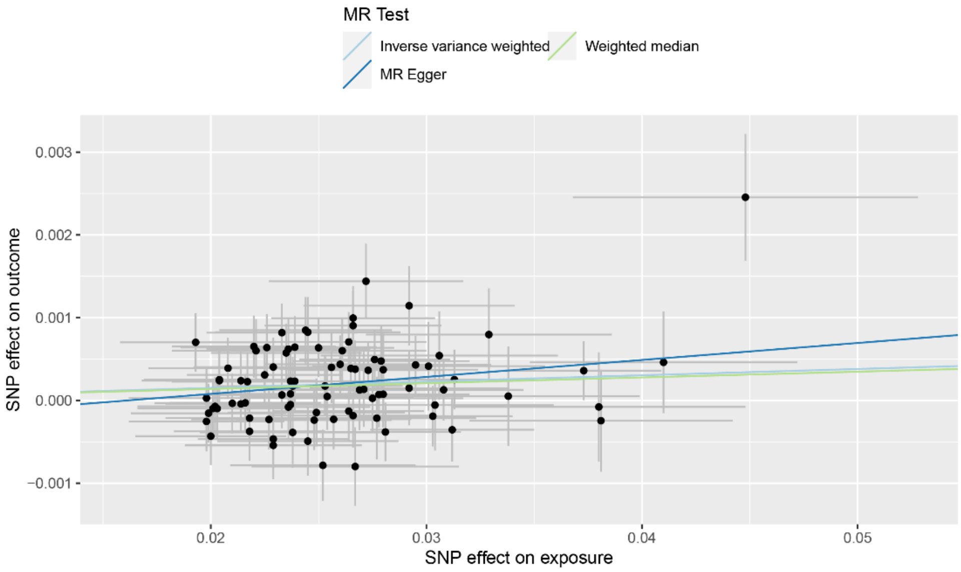

Supplementary Figure S5. Scatter plots for the causal association between depression and painful gums.

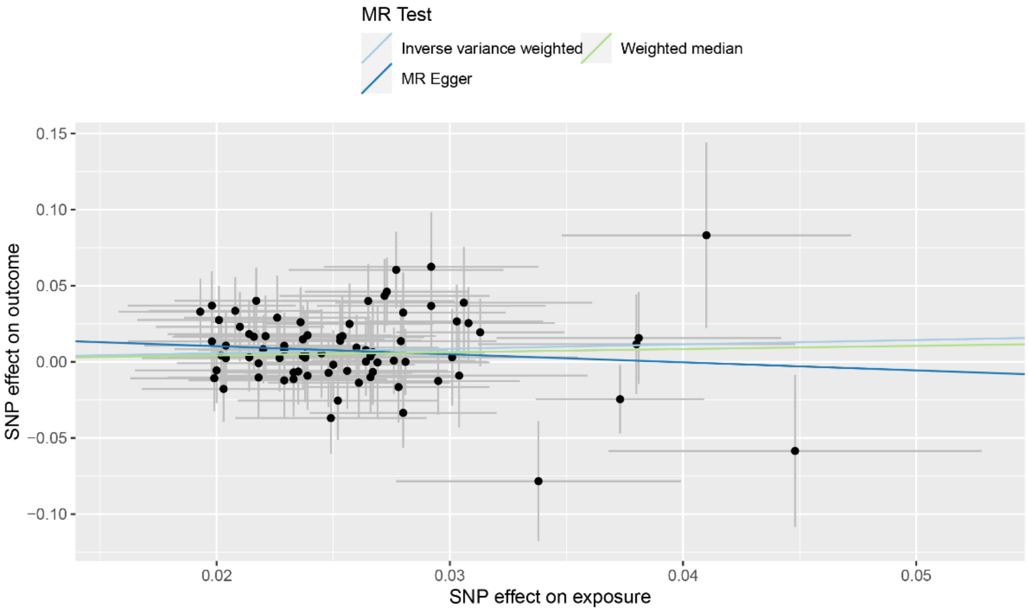

Supplementary Figure S6. Scatter plots for the causal association between depression and chronic periodontitis.

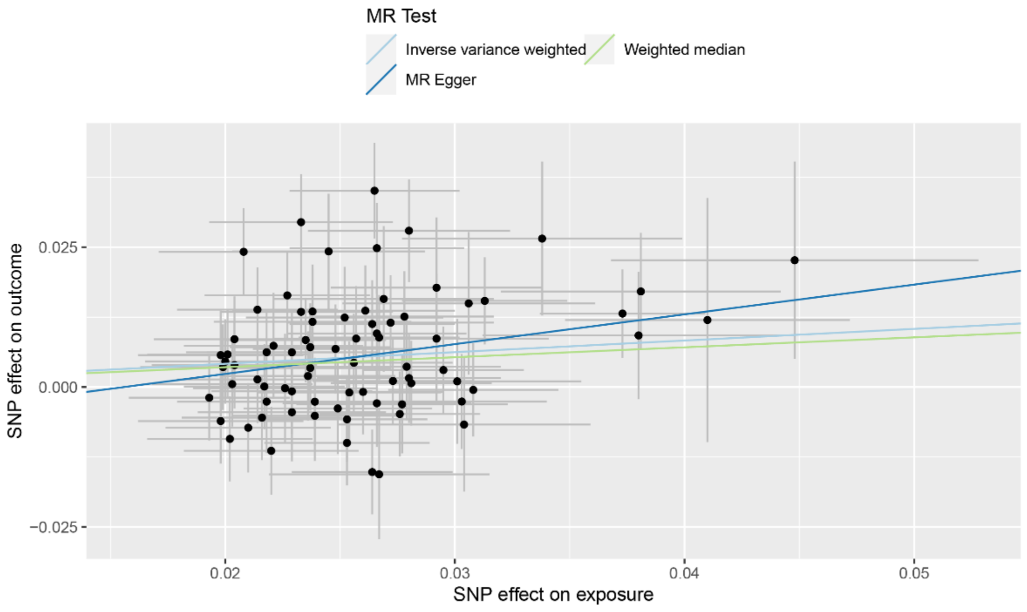

Supplementary Figure S7. Scatter plots for the causal association between depression and chronic diseases of tonsils and adenoids.

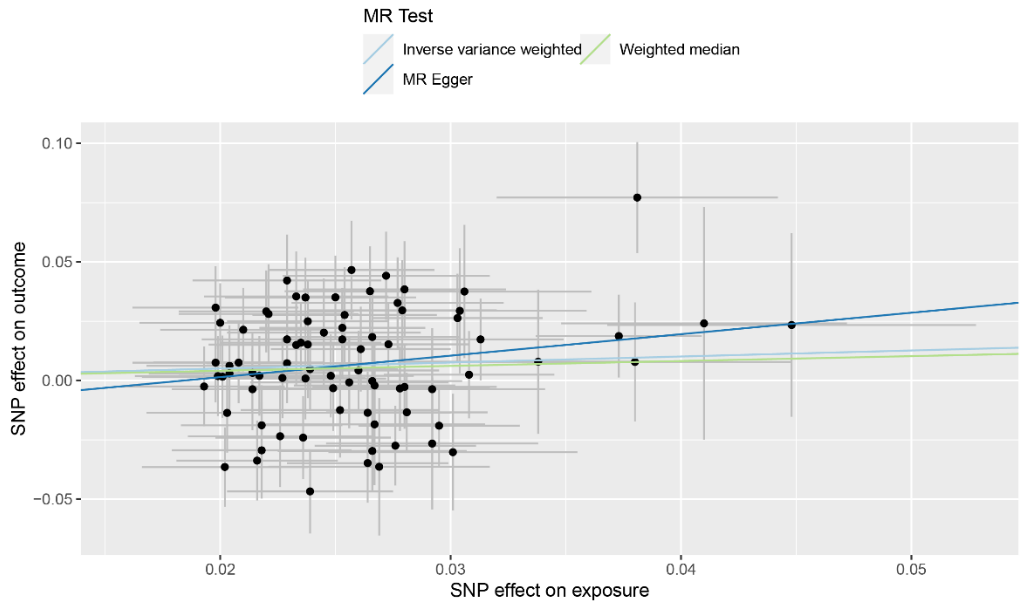

Supplementary Figure S8. Scatter plots for the causal association between depression and peritonsillar abscess.

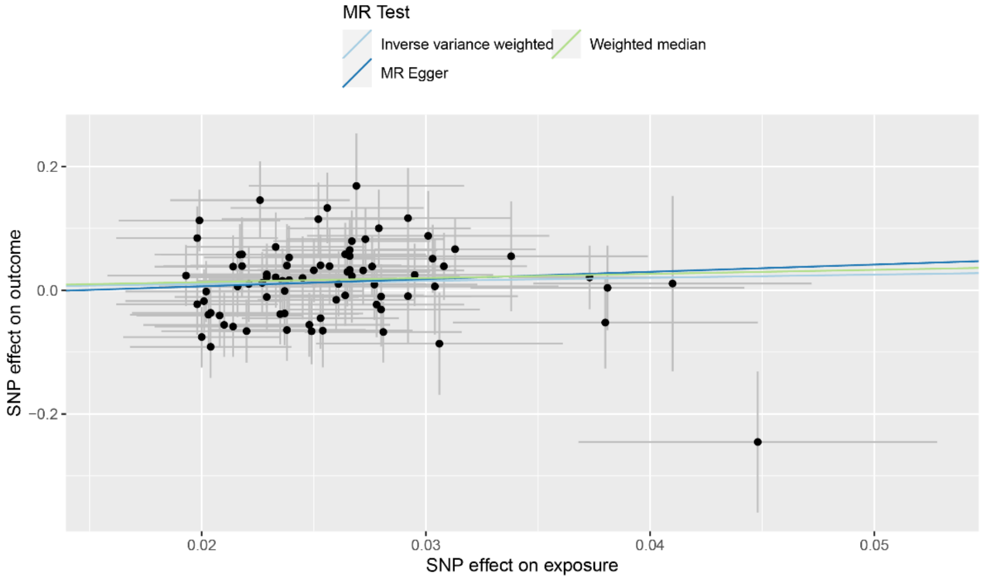

Supplementary Figure S9. Scatter plots for the causal association between depression and excessive attrition of teeth.

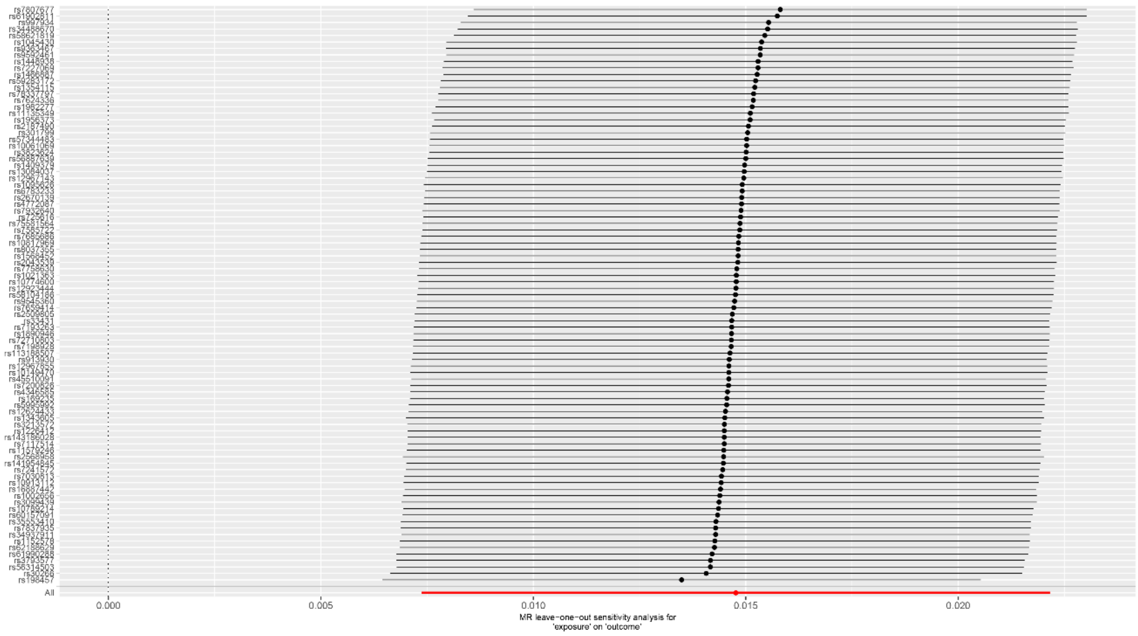

Supplementary Figure S10. Leave-one-out plots for the causal association between depression and mouth ulcers.

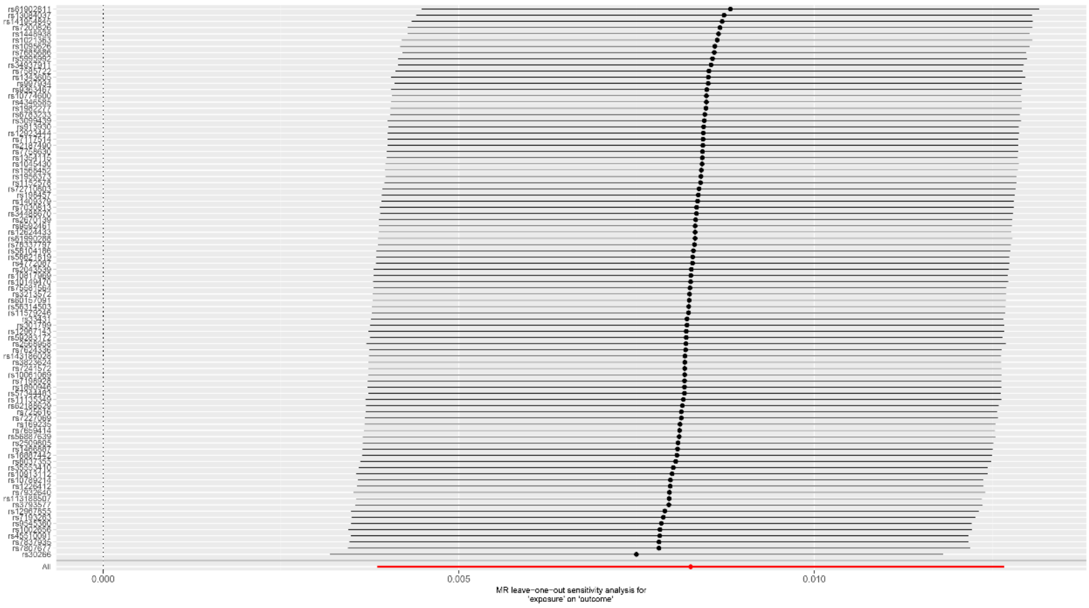

Supplementary Figure S11. Leave-one-out plots for the causal association between depression and toothache.

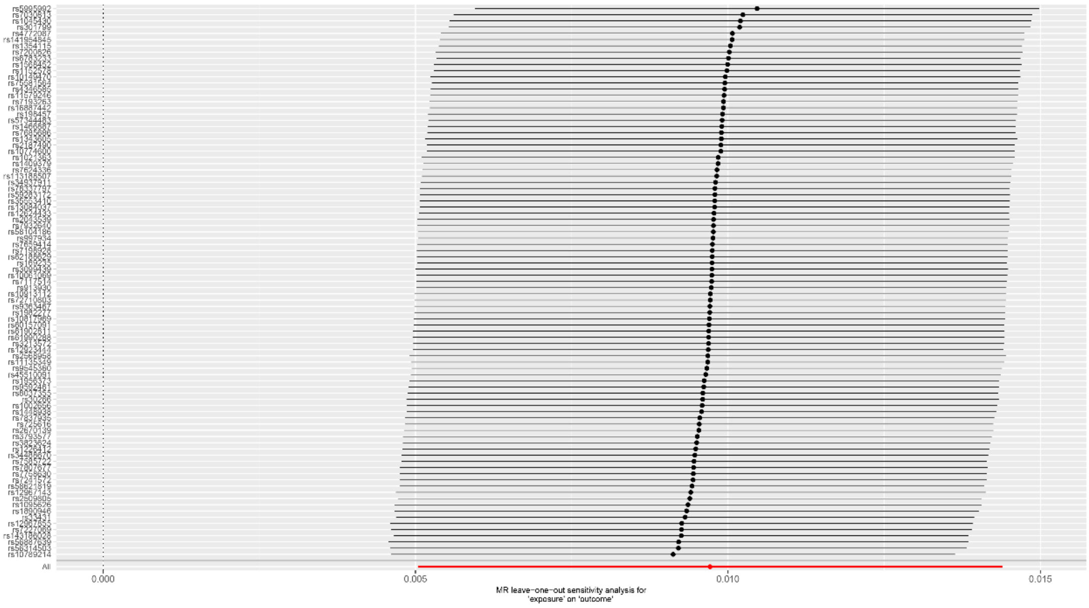

Supplementary Figure S12. Leave-one-out plots for the causal association between depression and loose teeth.

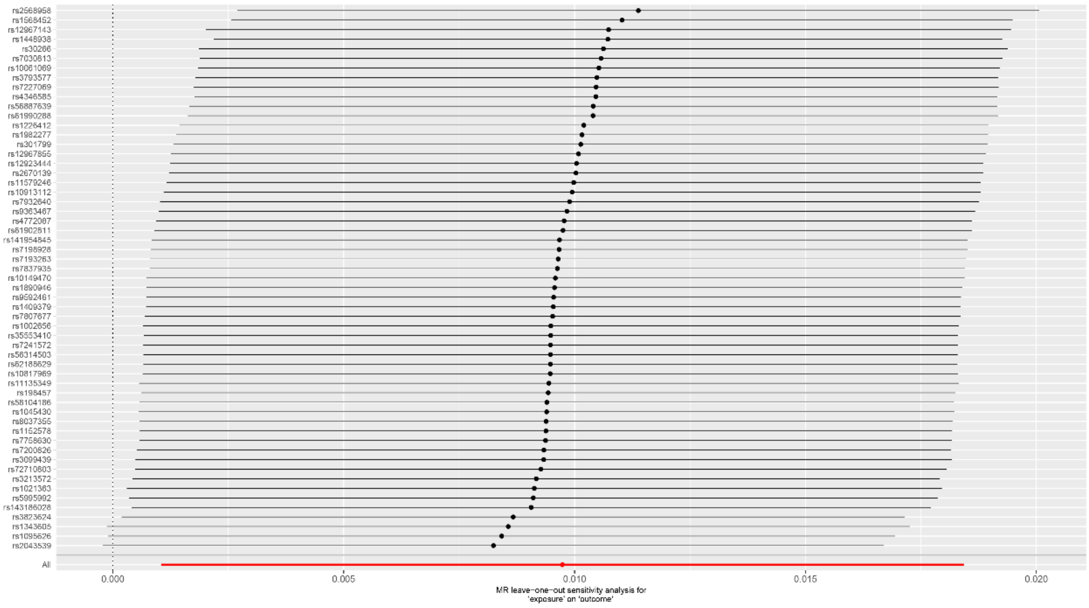

Supplementary Figure S13. Leave-one-out plots for the causal association between depression and bleeding gums.

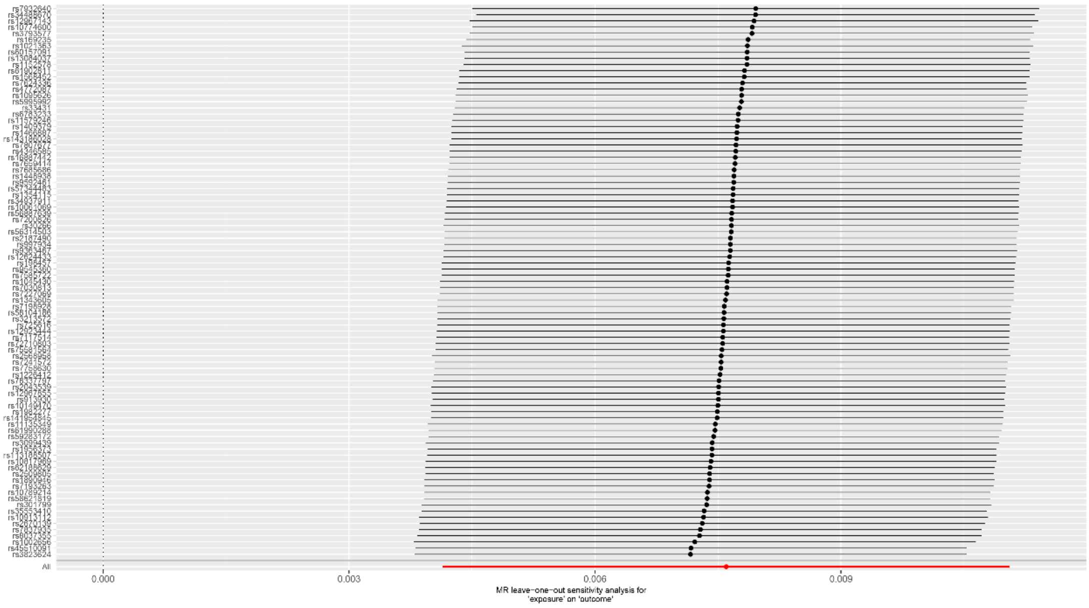

Supplementary Figure S14. Leave-one-out plots for the causal association between depression and painful gums.

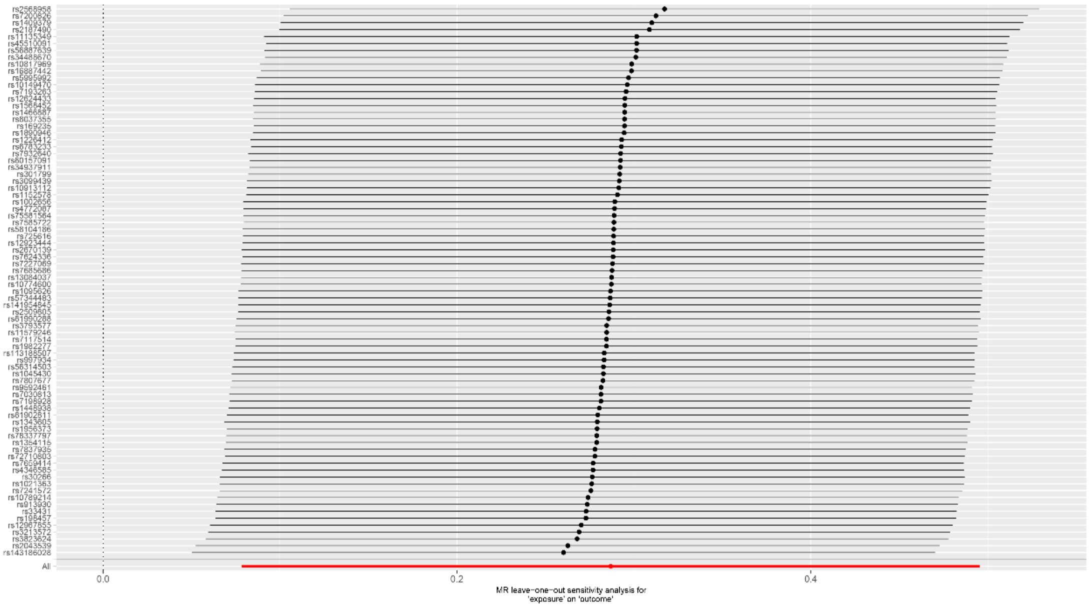

Supplementary Figure S15. Leave-one-out plots for the causal association between depression and chronic periodontitis.

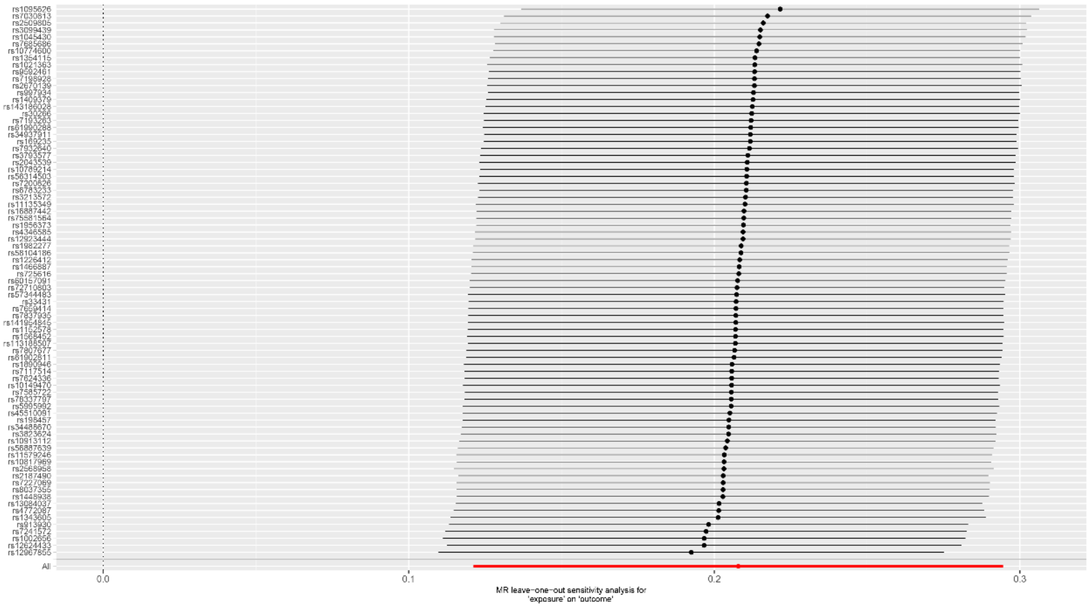

Supplementary Figure S16. Leave-one-out plots for the causal association between depression and chronic diseases of tonsils and adenoids.

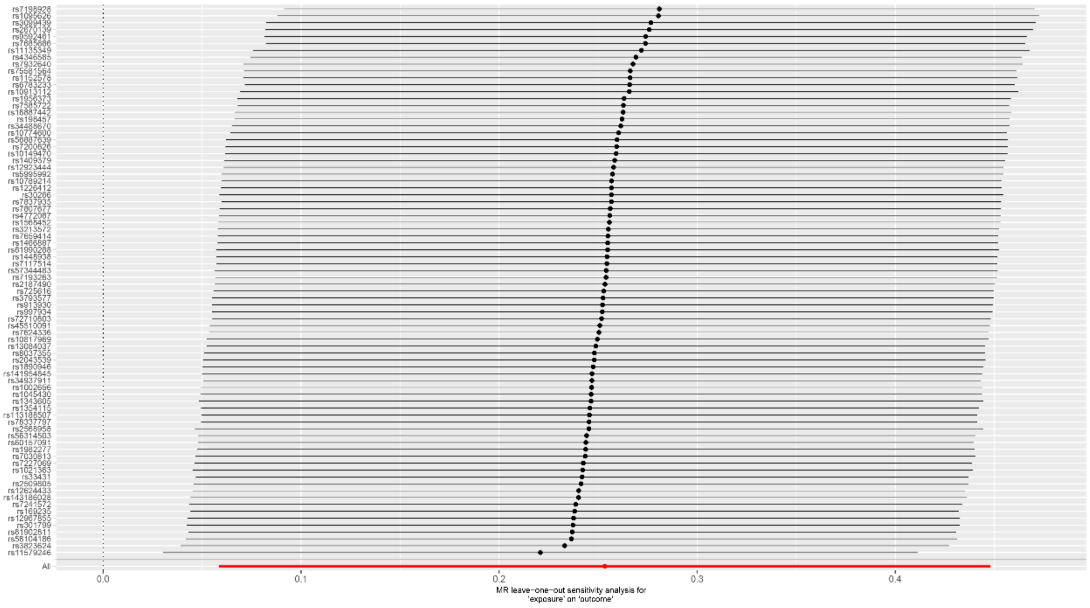

Supplementary Figure S17. Leave-one-out plots for the causal association between depression and peritonsillar abscess.

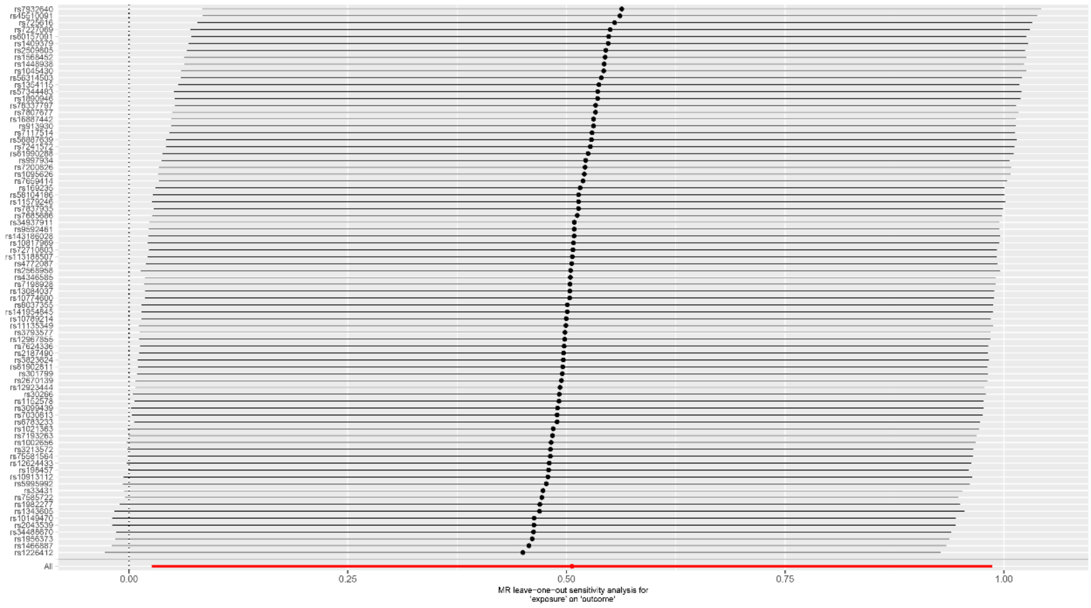

Supplementary Figure S18. Leave-one-out plots for the causal association between depression and excessive attrition of teeth.

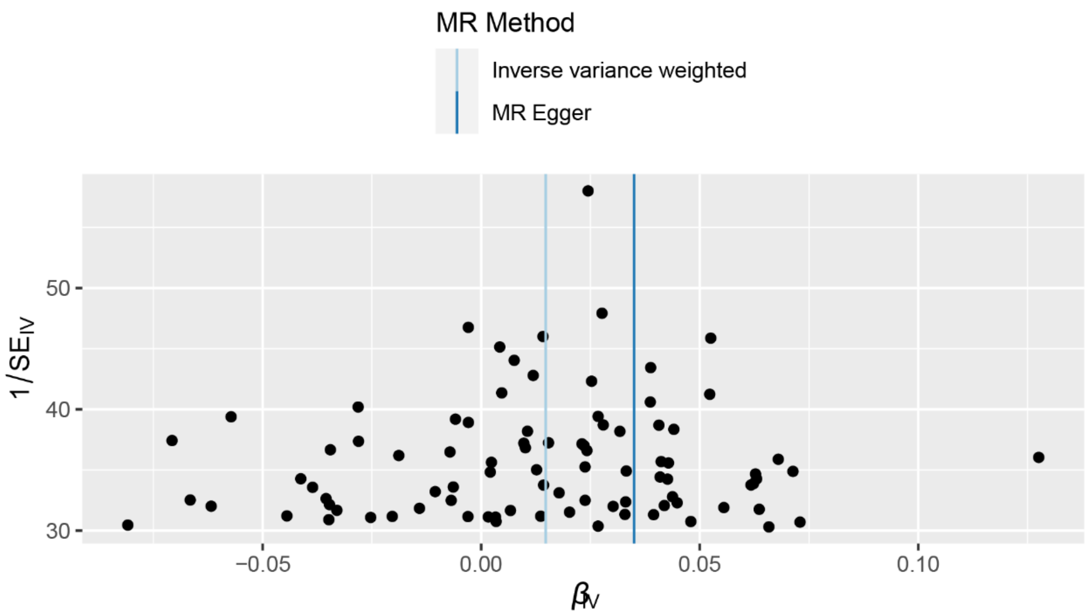

Supplementary Figure S19. Funnel plot for the causal association between depression and mouth ulcers.

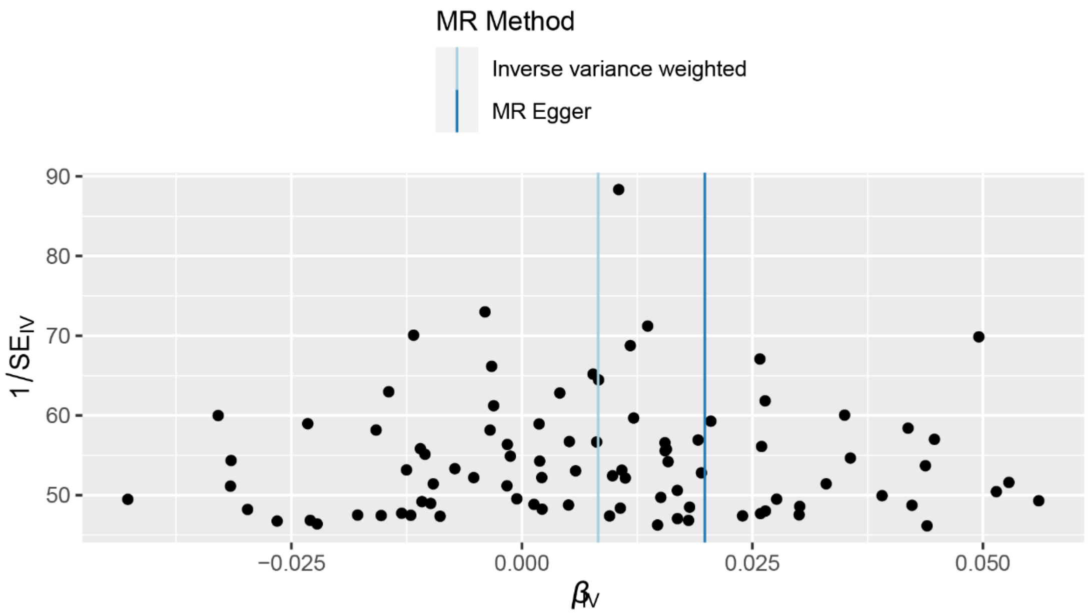

Supplementary Figure S20. Funnel plot for the causal association between depression and toothache.

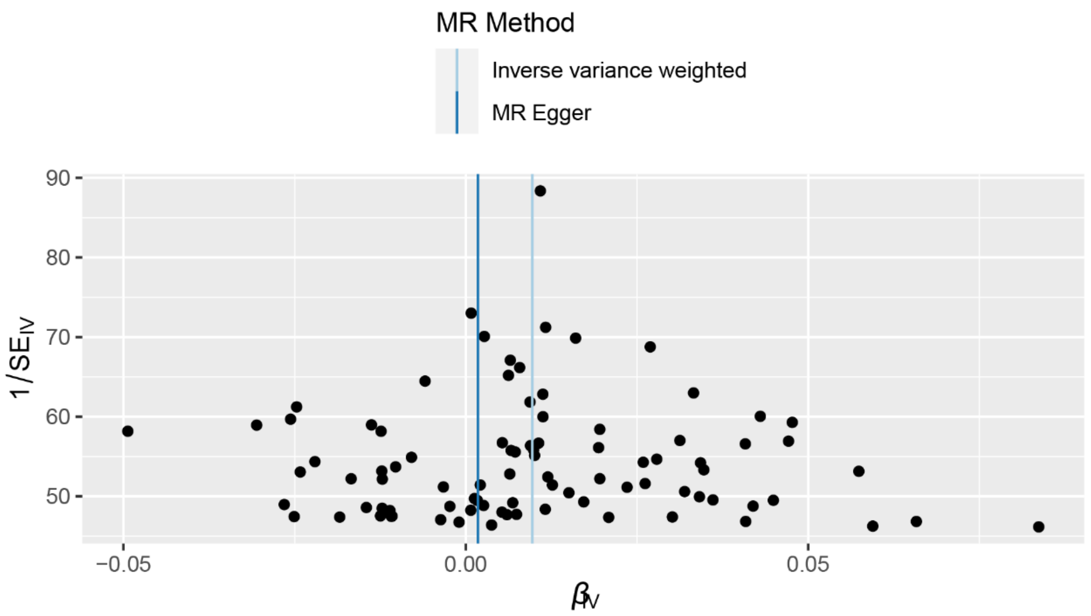

Supplementary Figure S21. Funnel plot for the causal association between depression and loose teeth.

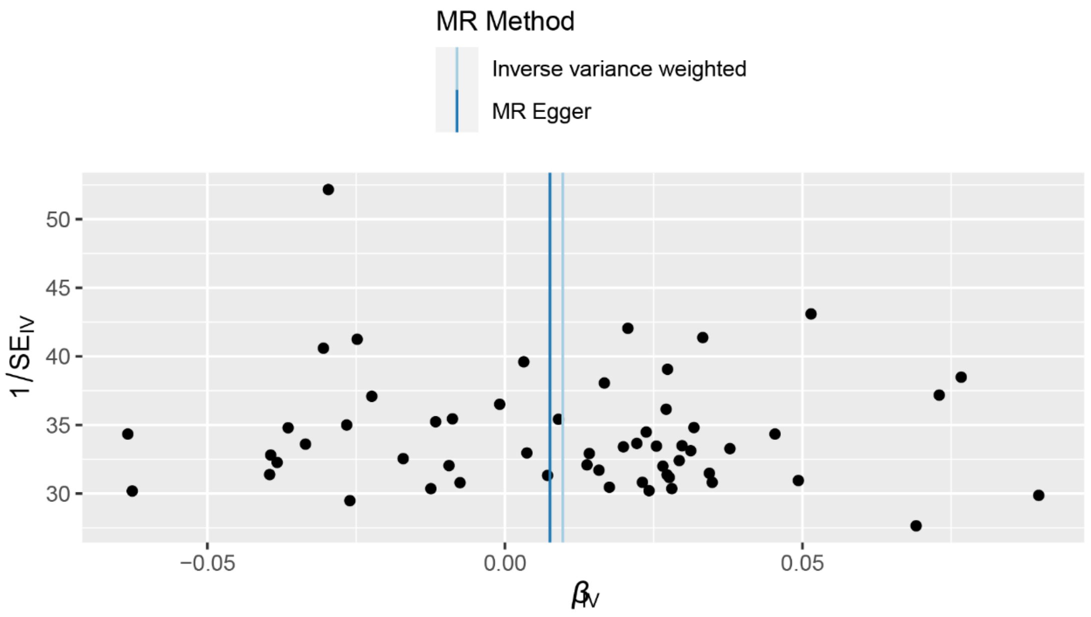

Supplementary Figure S22. Funnel plot for the causal association between depression and bleeding gums.

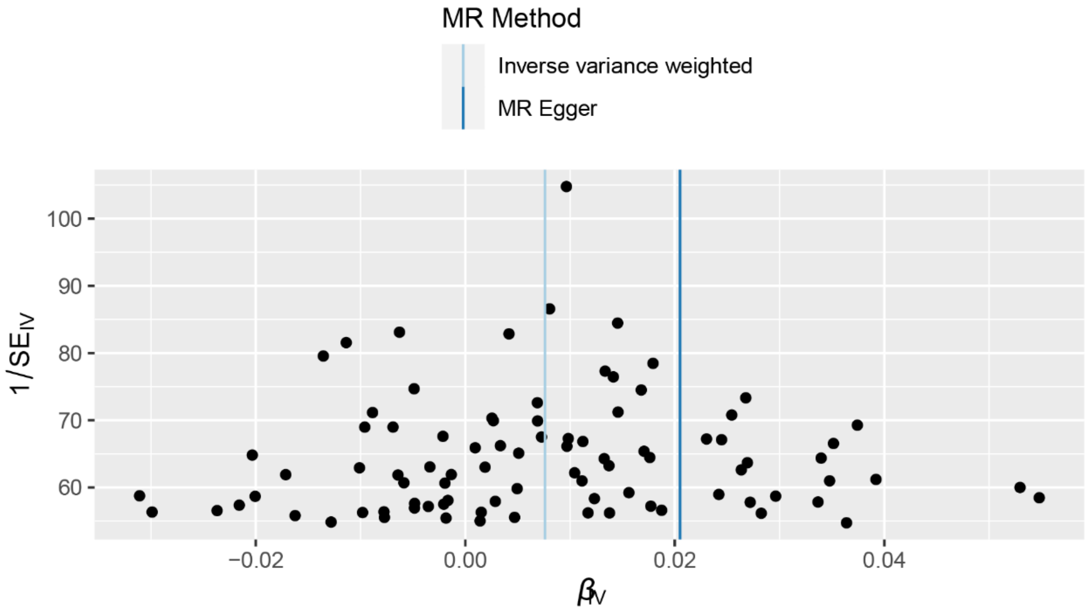

Supplementary Figure S23. Funnel plot for the causal association between depression and painful gums.

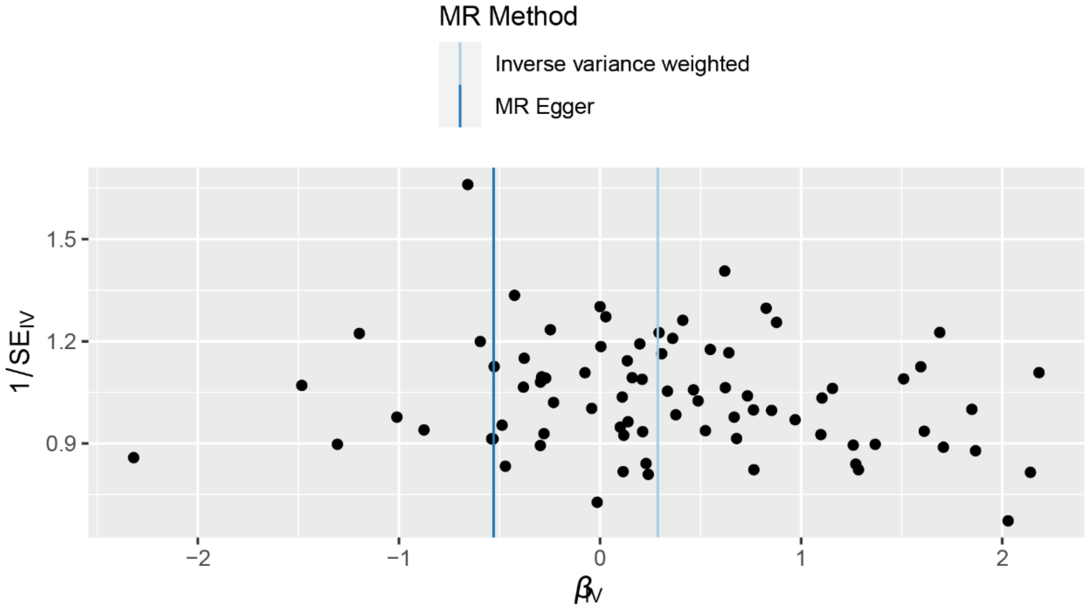

Supplementary Figure S24. Funnel plot for the causal association between depression and chronic periodontitis.

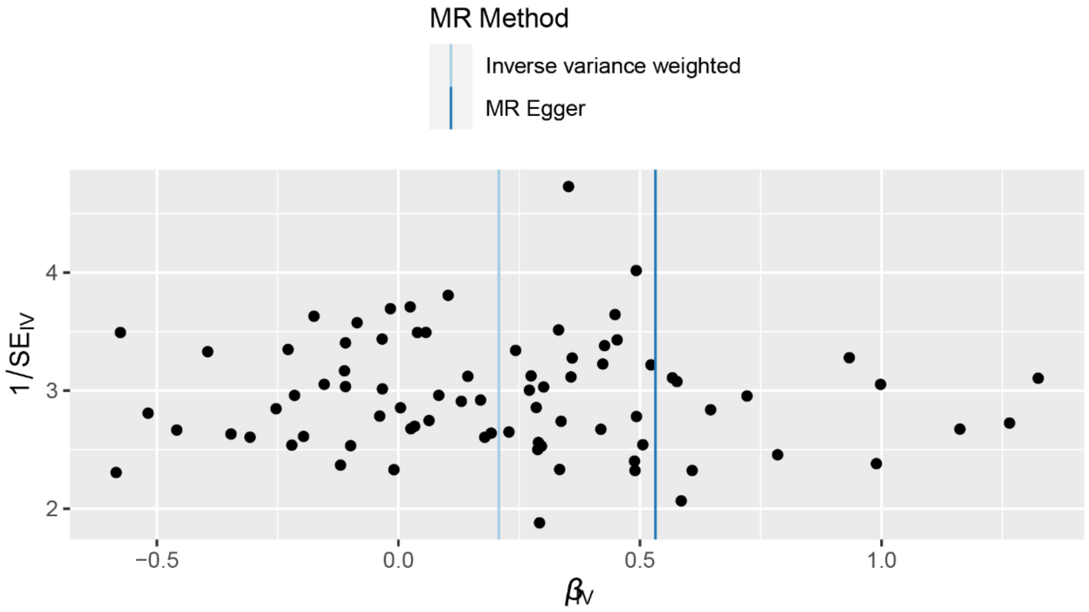

Supplementary Figure S25. Funnel plot for the causal association between depression and chronic diseases of tonsils and adenoids.

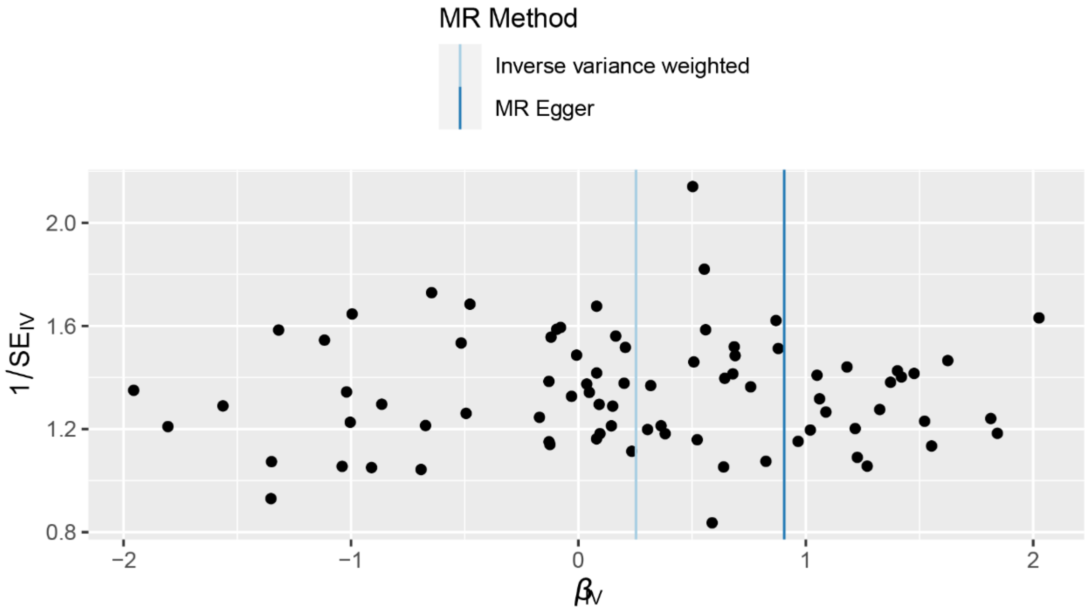

Supplementary Figure S26. Funnel plot for the causal association between depression and peritonsillar abscess.

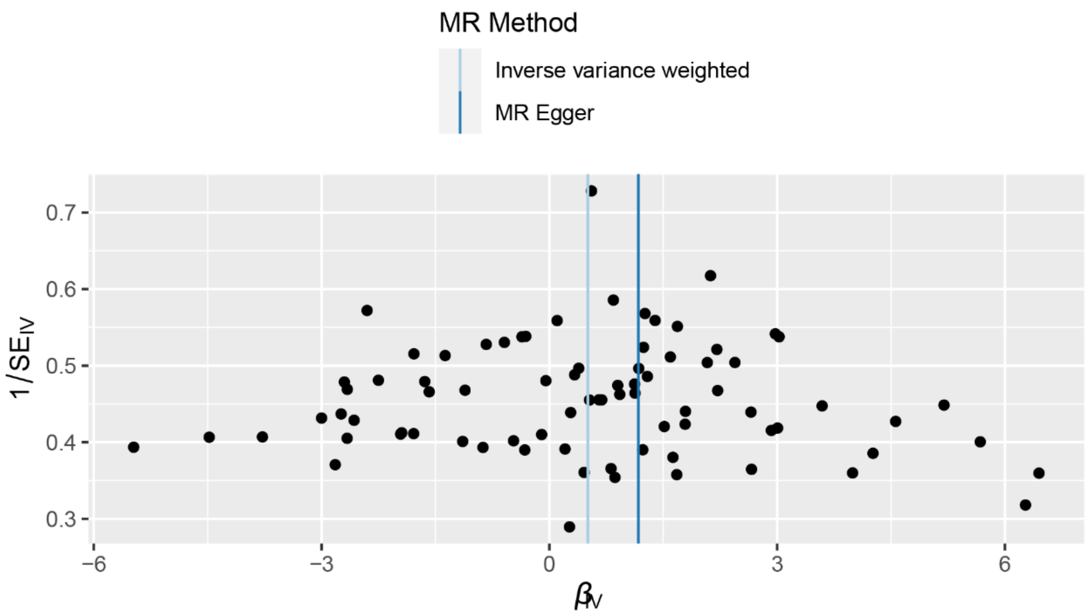

Supplementary Figure S27. Funnel plot for the causal association between depression and excessive attrition of teeth.
